# AI-driven precision diagnosis and treatment of prostate cancer: a narrative review

**DOI:** 10.3389/fonc.2026.1877570

**Published:** 2026-07-16

**Authors:** Tonghui Chu, Youzhao Zhang, Jianuo Du, Abudurexiti Mierxiati

**Affiliations:** 1 School of Gongli Hospital Medical Technology, University of Shanghai for Science and Technology, Shanghai, China; 2 Department of Urology, Pudong Gongli Hospital,Shanghai University of Medicine & Health Science, Shanghai, China

**Keywords:** artificial intelligence, diagnosis, precision medicine, prostate cancer, treatment

## Abstract

Prostate cancer is one of the most common cancers in men, and its incidence has been increasing annually. Early screening, accurate diagnosis, and personalized treatment are crucial for improving patient prognosis. With technological advancements, artificial intelligence (AI) has been increasingly applied as a key tool in the diagnosis and treatment of prostate cancer. In the field of diagnosis, AI-driven multi-modal models that integrate imaging, pathological, and clinical data have enabled precise segmentation and grading of prostate cancer. This not only enhances diagnostic accuracy and efficiency but also reduces differences in judgments between medical professionals. In treatment, AI has been integrated into surgical procedures, radiation therapy, and targeted drug development to optimize treatment plans and explore relevant biological indicators and treatment targets. Although various AI models have demonstrated clinical value, most have not been put into practical clinical use, which highlights the significant limitations of current AI technology. This review focuses on the practical application outcomes and future development directions of AI in prostate cancer diagnosis and treatment. This study aims to explore the potential of AI in clinical practice, promote its deep integration with clinical work, and construct reliable and safe diagnostic and therapeutic models. These efforts are expected to alleviate the workload of medical professionals, improve diagnostic accuracy, facilitate personalized treatment planning, and ultimately enhance patient prognosis and quality of life. The trend of intelligent medicine should be embraced, and AI-assisted diagnostic and therapeutic technologies should be actively and rationally adopted to promote the advancement of medical care.

## 1 Introduction

Prostate cancer is one of the most common cancers affecting the genitourinary system and ranks among the most prevalent cancers in men. According to statistics from 2022, 1.5 million new cases and 397,000 deaths related to this disease are recorded globally. This makes prostate cancer the second most common cancer worldwide and the fifth leading cause of cancer-related death in men (1). The latest projections from the United States suggest that prostate cancer may become the most common cancer among American men and the second leading cause of cancer death in the country (2). With the accelerating aging of the population, the incidence of prostate cancer continues to rise each year.

Prostate cancer develops quietly and progresses slowly, so early detection is often challenging. When tumor enlargement compresses the urethra and causes symptoms of lower urinary tract obstruction, the condition is often confused with benign prostatic hyperplasia (3). Given the high incidence and mortality of prostate cancer, early screening, accurate diagnosis, and precise personalized treatment plans are crucial for significantly reducing the physical and psychological burden on patients.

**Figure 1:**
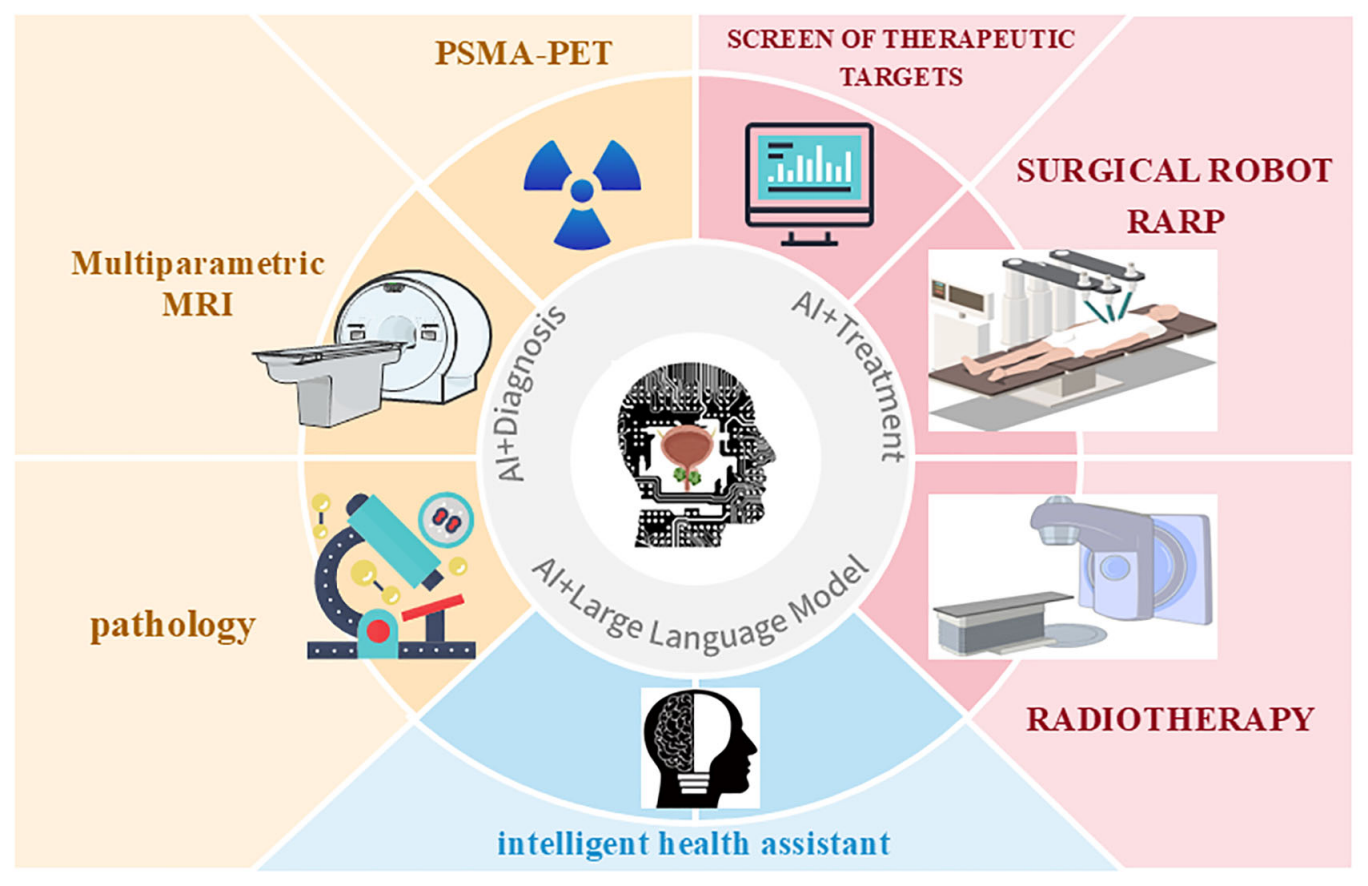
At present, the main application of artificial intelligence in the diagnosis and treatment of prostate cancer, Created with MedPeer (medpeer.cn).

Artificial intelligence (AI) is a scientific technology that uses computers to simulate intelligent human behavior. AI methods are generally divided into two main types: machine learning (ML) and deep learning (DL). In particular, DL based on neural network structures has been widely applied in the medical field. Success has been achieved through its use in image registration, feature extraction, detection of anatomical and cellular structures, tissue segmentation, computer-assisted disease diagnosis, and prognosis prediction (4). The rapid advancement of AI in healthcare provides promising solutions for improving diagnostic accuracy and efficiency, especially in the field of prostate cancer. The emergence of AI is expected to fundamentally change the clinical practice of medical professionals (5). Currently, AI models are being applied in both the diagnosis and treatment of prostate cancer. They enhance detection rates, enable precise staging and grading, and improve outcomes by analyzing pathological slides and radiomic data. Additionally, they predict complications following radical prostatectomy or radiation therapy, as well as survival outcomes. Leveraging AI’s ability to sift through vast amounts of information, these models identify potential biomarkers and therapeutic targets. Large Language Models (LLMs) assist in the automated generation of reports and the extraction of key information from them, enabling the dissemination of prostate cancer knowledge to patients in a more accessible and comprehensive manner. (**Figure 1**).

## 2 Methods

### 2.1 The review objective

This manuscript is a narrative review that systematically summarizes the latest applications of AI in the diagnosis and treatment of prostate cancer, covering imaging evaluation, pathological examination, radical prostatectomy, radiotherapy, Biomarker screening and therapeutic targets, as well as the implementation of LLMs in this field. The literature retrieval and screening procedures were performed following the Preferred Reporting Items for Systematic Reviews and Meta-Analyses (PRISMA) guidelines; nevertheless, this work does not constitute a formal systematic review.

### 2.2 Data sources and search strategy

A literature search was conducted in PubMed and Web of Science to screen English original research articles published between January 2022 and June 2026 that focused on the application of AI in the diagnosis and treatment of prostate cancer. The search keywords were prostate cancer, artificial intelligence, machine learning, deep learning, multiparametric magnetic resonance imaging (MP-MRI), PET/CT, digital pathology, multimodal diagnosis, radical prostatectomy (RP), radiotherapy, endocrine therapy, and LLMs. Considering the rapid development of AI research in oncology, the eligible English original articles were enrolled for the construction of this narrative review.

### 2.3 inclusion and exclusion

**Figure 2:**
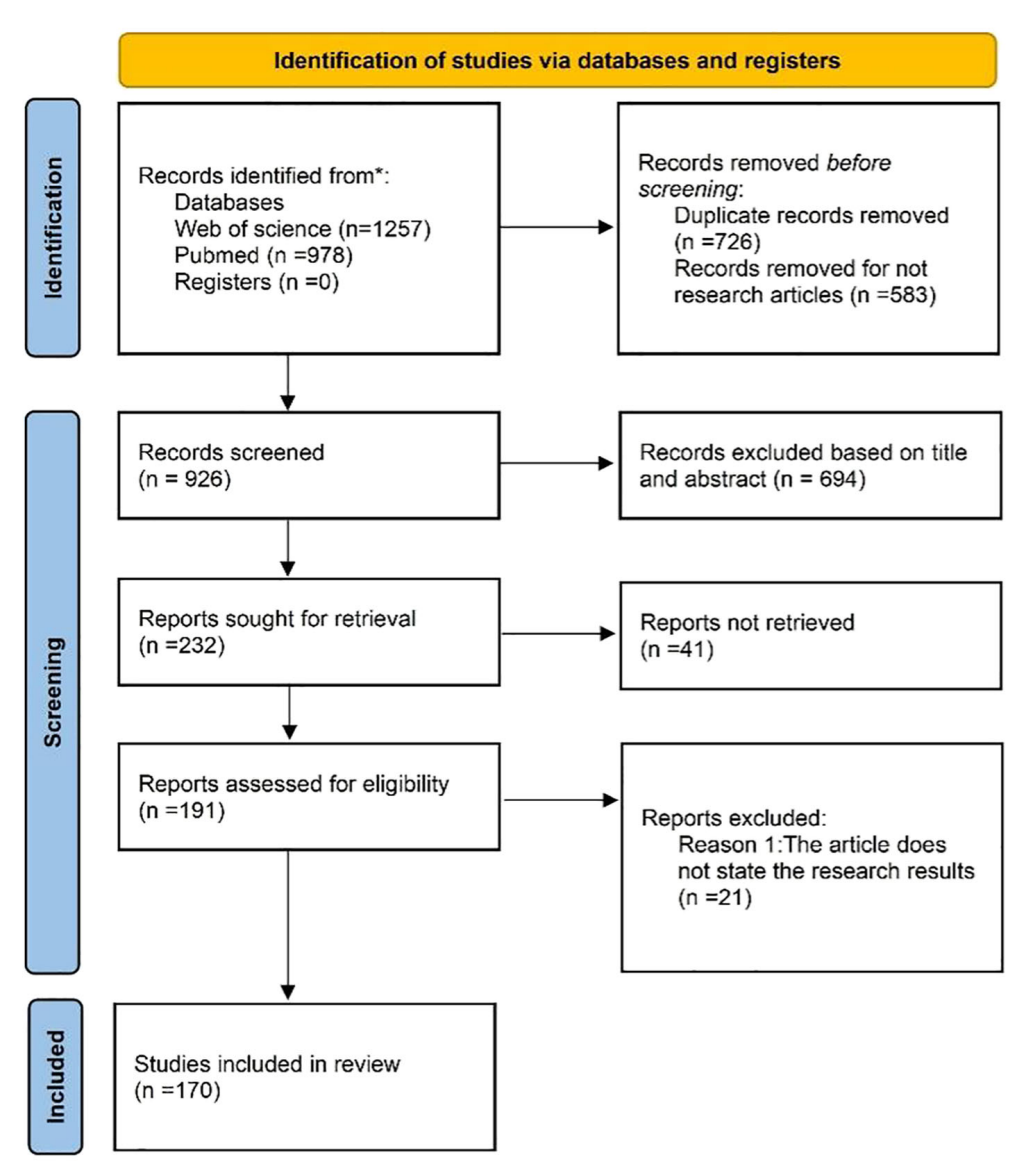
PRISMA flow diagram of the literature search and selection process. The diagram illustrates the number of records identified from PubMed and web of science, screened, and finally included.

Original research articles and high-impact factor systematic reviews published within the past five years were eligible for inclusion. Exclusion criteria were predefined as follows: editorials, letters to the editor, conference abstracts/proceedings, case reports, publications with inaccessible full-text versions, studies that failed to clearly report core research outcomes, and non-English articles (**Figure 2**).

## 3 Application of AI in prostate cancer diagnosis

### 3.1 MRI + AI for prostate cancer detection

In the early stage of MRI, the diagnosis of prostate cancer was primarily based on single-parameter scans (T1WI and T2WI),which have low sensitivity and specificity, leading to frequent missed diagnoses of clinically significant prostate cancer (CS-PCa).This issue is partly caused by technical constraints: low-field MRI systems cannot clearly distinguish prostate regions, resulting in inaccurate tumor localization and higher misdiagnosis rates (6, 7). Moreover, there is an urgent need for quantitative and practical tools in medical imaging diagnosis. As MP-MRI has become more widely used and with the standardization of prostate MRI interpretation through the Prostate Imaging Reporting and Data System (PI-RADS), the detection of CS-PCa has increased, reducing unnecessary biopsies and effectively curbing overtreatment. However, manual PI-RADS scoring is inconsistent due to differences in diagnostic skills and experience among different professionals. Furthermore, the diagnostic accuracy for PI-RADS grade 3 lesions (often referred to as the “gray zone”) is only 30–40%, with a high rate of misclassification between benign and malignant lesions (8–10).

The convergence of AI and imaging aims to develop AI algorithms capable of automatically analyzing MRI images to improve image quality, diagnostic accuracy and specificity, automatically measure the extent of cancerous lesions with high precision, reduce unnecessary examinations, and minimize inter-radiologist variability (11). In the field of lesion segmentation, the ProGNet model proposed by Soerensen accurately segments prostate lesions and has been integrated into clinical workflows; studies show that ProGNet’s segmentation performance is significantly superior to that of radiographers (DSC = 0.92 ± 0.02 vs. 0.89 ± 0.05) (12).The DL-CAD system (13)was shown to improve the diagnostic accuracy for CS-PCa (PI-RADS ≥ 4) from 0.84 to 0.88, while the average review time was reduced by 21%. This result highlights the value of AI in improving efficiency and reducing subjective variability. Most AI algorithms are models with unclear decision-making processes, which remains a persistent challenge for clinicians (14). To solve the problem of poor interpretability of AI models, Hamm,et al. (15) proposed an interactive explainable AI (XAI) model for classifying and interpreting CS-PCa. High diagnostic accuracy was achieved by this model on both internal (AUC = 0.89) and external (AUC = 0.87) test sets. XAI generates three-dimensional color heatmaps on MRI images, highlighting tumor regions predicted by the model and providing textual explanations. Expert validation confirmed that the textual explanations reached an accuracy of 80% (1080/1352).

In recent years, a large number of similar studies have confirmed that AI models have reached or exceeded the performance of radiologists in CS-PCa detection, precise grading, screening, and prognostic prediction (**Table 1**). The integration of increasingly sophisticated DL models with MP-MRI image features has reduced inter-observer variability and improved diagnostic accuracy. In particular, they have improved the diagnostic accuracy and specificity of CS-PCa, predicted poor pathological responses, and precisely segmented cancerous lesions, demonstrating detection capabilities comparable to or even superior to those of radiologists. However, as most models are based on equipment from a single manufacturer, the accuracy of lesion detection and PI-RADS scoring declines once scanning parameters are altered; furthermore, for complex anatomical structures, the models’ segmentation and lesion recognition capabilities drop precipitously. When dealing with micro-lesions and micro-invasion, AI exhibits poor diagnostic stability, and the accuracy of overall lesion segmentation is significantly lower than that of prostate gland segmentation. Future efforts should be based on standardized databases, with a focus on overcoming the identification of PI-RADS grey areas. There is a need to develop highly interpretable AI models and visualize the AI analysis process for prostate cancer, thereby enhancing the confidence of clinicians and patients in the results.

**Table 1:** Primary outcomes and objectives of AI-assisted MP-MRI models for prostate cancer diagnosis in the past three years.

Study(author, year)	AI model and key advantages	Main finding	Data
Huang,2025 (16)	DL(GAN) Reduce the potential allergic reactions, toxicity of gadolinium agent, and reduce the burden of patients.	• The structural similarity between simulated enhanced images and real enhanced images is 0.82, 0.71, and 0.69 on the and two external test sets, respectively. • Three radiologists showed agreement of Cohen k = 0.96 when scoring PI-RADS using simulated or real images.	Training Set:244 Internal Test Set:104 External Test Set1:143 External Test Set2:76
Giganti,2025 (17)	DL-CAD Automatically performs prostate segmentation and initial lesion screening, outputs a 1–5 risk score, and demonstrates generalized performance validated across multiple centers, brands, field strengths, and scanner models.	• The diagnostic accuracy of the Pi AI model (AUC = 0.91) is comparable to that of experienced radiologists (AUC = 0.95). • The sensitivity and specificity of the Pi AI model are 95% and 67%, respectively, results that are close to those of physicians (99%, 73%).	Training Set:793 Testing Set:252
Roest,2023 (18)	DL(3D U-Net)- Using serial prostate MRI images to enhance the detection accuracy of clinically significant prostate cancer outperforms traditional single-scan analysis and radiologist assessment.	• The AI monitoring model demonstrated significantly higher diagnostic performance for clinically meaningful prostate cancer when analyzing both “previous” and “current” consecutive scans (AUC increased from 0.73 to 0.81, p=0.04). • The optimal AI monitoring model (combining sequential MRI features and clinical parameters) demonstrated significantly superior diagnostic accuracy compared to evaluation by experienced radiologists (AUC 0.86 vs. 0.69, p=0.02).	Training Set:1291 Testing Set:143
Engel, 2025 (19)	DL(CNN) While pursuing high sensitivity, enhance specificity and reduce unnecessary needle biopsies.	• When PI-RADS ≥ 4, the sensitivity was lower than that of physicians (89.6% vs. 95.6%), but the specificity and overall accuracy were significantly higher than those of physicians (57.7% vs. 31.4%, 73.5% vs. 63.2%).	Testing Set:272
Umapathy, 2025 (20)	DL(RL) Effectively providing additional decision-making information for PI-RADS=3 cases has significantly reduced unnecessary needle biopsies while maintaining a high detection rate for CS-PCa	• The AUC for the full PI-RADS scoring group was 0.88, significantly higher than the radiologist's AUC of 0.78. • In the gray zone with PI-RADS 3.41% of benign biopsies were avoided. When combined with clinical data, the proportion of avoided biopsies increased to 46%.	Training Set:21938 Testing Set:764
Yuan,2025 (21)	DL(CNN) The Z-SSMNet model was developed by integrating multidimensional convolutions and self-supervised learning, achieving high-precision detection and diagnosis of CS-PCa	• Achieved the top ranking in the PI-CAI Phase 1 test set AUC = 0.881; ranked second in the final, larger-scale test set AUC = 0.909.	Training Set:10000+ Testing Set:1000
de Almeida,2025 (22)	ML This study confirms the feasibility of automatically identifying prostate MP-MRI sequence types based on DICOM metadata, which is crucial for data preparation prior to training clinical AI models.	• Nearly perfect differentiation of five prostate MRI sequence types, with F1-scores consistently above 0.97 across the test set • Model performance stabilizes after training on just 2–10% of the total dataset.	Testing Set:7891
Yang,2024 (23)	DL(nnU-Net) Validating the independent prognostic value of AI-based tumor volume segmentation (VAI), which demonstrates superior predictive efficacy compared to traditional clinical staging, holds promise for establishing a noninvasive prognostic prediction tool.	• For every 1 ml increase in tumor volume, the risk of metastasis increased by 9% in the radiotherapy group and by 22% in the RP group. • The AUC for metastasis prediction over seven years was 0.84, higher than the AUC of 0.74 for NCCN staging.	Training Set:288 Testing Set:radiotherapy group:150RPgroup:294
Li,2024 (24)	DL(CUT+U-Net) Effectively overcomes model performance degradation caused by variations in MRI acquisition parameters, eliminating the need for separate model training and achieving high computational efficiency.	• For with PI-RADS ≥ 3, the AUC increased to 0.79. • Under unfavorable ADC b-value settings, the AUC also improved to 0.76 compared to the baseline model.	Training Set:3458
Cai,2024 (25)	DL(CNN) Develop a fully automated DL model requiring only clinically meaningful prostate cancer labels (without tumor location annotations), enabling unsupervised tumor localization through GRAD-CAM.	• DL models perform comparably to experienced radiologists in detecting CS-PCa (AUC 0.89: 0.89). • Using Grad-CAM visualization, the model localizes CS-PCa lesion regions. Localization success rates reached 92% and 97% in internal and external test sets, respectively.	Training Set:5035 Internal Test Set:400 External Test Set:204
Gu,2023 (26)	DL Based on pretreatment MRI images, a novel DL network NAF-Net was utilized to develop an integrated tool (DL-nomogram) that accurately predicts the risk of adverse pathological events (AP) and biochemical recurrence (BCR) following radical prostatectomy in prostate cancer patients.	• The NAF-Net model outperformed ResNet50 in predicting adverse pathology (AUC: 0.799 vs. 0.703). • The DL-nomogram demonstrated superior performance in predicting AP and hemicBCR.	Training Set:294 Internal Test Set:73 External Test Set:147
Karagoz,2023 (27)	DL(nnU-Net) Evaluate the effectiveness and generalization capability of DL models trained on large-scale datasets for detecting CS-PCa.	• On the validation set and test set, the model achieved AUC values of 0.888 and 0.889, respectively, with average precision values of 0.732 and 0.614.	Training Set:1476 Internal Test Set:1002
Sun,2023 (28)	DL To compare the differences in diagnostic performance, reading time, and diagnostic confidence between independent interpretation by radiologists and AI-assisted interpretation for MRI-detectable CS-PCa; and to validate the practical clinical value of this AI software in real-world, multicenter, and multi-device settings.	• AI assistance significantly increased the sensitivity of physicians detecting CS-PCa lesions from 40.1% to 59.0%. • AI assistance significantly increased the specificity of physician diagnoses from 57.7% to 71.7%. • AI assistance substantially reduced the median reading time from 423 seconds to 185 seconds.	External Test Set:480
Bardis,2021 (29)	DL(U-Net) Develop a DL model capable of automatically and accurately segmenting the transition zone and peripheral zone of the prostate to assist in PI-RADS scoring.	• The model can delineate the prostate contour with exceptional precision (ADC = 0.940). The transition zone exhibits an ADC value of 0.910. • Model processing is exceptionally fast, with segmentation of a single image completed within one second.	Training Set:145 Testing Set:48
Kim,2023 (30)	DL(CNN) Evaluate the performance of MP-MRI sequence combinations in detecting CS-PCa using 3D CNNs. Visualize decision-making rationale through heatmaps and correlate with Gleason scores to enhance physician confidence.	• Best-performing model: T2-ADC-DWI combination, achieving an AUC of 0.90 on the validation set and 0.89 on the independent test set. • Among single sequences, DWI and ADC sequences performed best (AUC = 0.84).	Training Set:204 Testing Set:140

### 3.2 PSMA-PET+AI for molecular-level diagnosis and metastasis detection of prostate cancer

Prostate-specific membrane antigen (PSMA) positron emission tomography (PET) has been recognized as a key tool in prostate cancer diagnosis. Conventional PET tracers target metabolically active cancer tissues; however, owing to the generally low metabolic activity of primary prostate cancer lesions, low sensitivity and resolution have been observed in the detection of primary tumors with these methods (31). PSMA is a type of transmembrane protein that is highly expressed in prostate cancer cells, with levels far exceeding those in other normal tissues (32). Its high specificity has significantly improved the detection sensitivity for both primary and metastatic prostate cancer lesions, indicating a qualitative improvement in accuracy compared with traditional techniques.

However, several clinical challenges are still faced by PSMA PET. First, internationally agreed-upon image evaluation standards are lacking—for example, criteria such as PSMA-RADS or PROMISE have not been widely adopted—hindering the comparison of interpretation results across different medical institutions. Second, lesion identification and quantitative analysis rely heavily on physician experience, with differences in judgments between observers reaching approximately 20% (33). Finally, the sensitivity for detecting low-uptake lesions (such as prostate cancer lesions that no longer respond to hormone therapy and do not express PSMA) remains insufficient, and the detection rate for small metastases (smaller than 5 mm) is limited (34). Novel technical solutions to these challenges have been provided by the application of AI in PSMA PET.

A predictive model for detecting invisible primary lesions was developed by Yi,et al. (35). When standard and delayed PET images were combined for prediction, an AUC of 0.925 was achieved, along with favorable sensitivity and specificity. This model not only overcomes the limitations of traditional imaging in detecting hidden lesions but also provides objective quantitative standards for clinical decision-making. A DL-based model for automatically classifying suspicious or nonsuspicious lesions was developed by Li (36), with an accuracy of nearly 0.8 achieved on both internal and external test sets. This high-performance classification model enables timely further examinations and treatment for suspicious lesions while avoiding overtreatment and reducing the burden on patients with nonsuspicious lesions.

An AI model was employed by Lindgren (37) to analyze PET–CT images for bone metastasis assessment, and the results revealed that its sensitivity was comparable to that of senior physicians (85.9% vs 83.8%). The calculated PET indicators exhibited moderate to strong correlations (r=0.65--0.72) with physician scores, providing an automated solution to address subjective variability in the quantitative analysis of bone metastases. A fully automated Deep-SSTL model was developed by Leung (38), achieving a 0.77 positive rate in detecting high-risk patients and 83% accuracy in risk stratification. Its unique feature lies in the automation of whole-body tumor burden quantification, and its performance continues to improve with the accumulation of data, demonstrating the evolutionary potential of “learning medical devices.”

The integration of pre-treatment and post-treatment PSMA PET imaging was pioneered by Cao,et al (39) to predict two-year metastasis-free survival (MFS) in patients with oligometastatic castration-sensitive prostate cancer (om-CSPC). An accuracy of 74% and an AUC of 0.76 were achieved by this model. The predictive performance of the model was further enhanced by incorporating clinical information (80% accuracy; AUC = 0.82), providing a prospective predictive tool for personalized treatment decisions.

The integration of PSMA-PET with AI has overcome technical bottlenecks in molecular imaging analysis, demonstrating unique advantages in the detection of metastatic lesions, quantification of tumor burden, and prognosis prediction. More importantly, AI overcomes the limitations of traditional imaging technologies, significantly improving the accuracy of identifying extracapsular invasion, seminal vesicle invasion, and biochemical recurrence; it enables tumor segmentation and localization at the microscopic level, effectively reducing the subjective bias inherent in traditional manual diagnosis (**Table 2**). However, numerous challenges inherent to PSMA-PET—such as a wide range of lesion uptake values, physiological uptake effects from adjacent organs, and image artifacts—complicate the development of models with strong generalization capabilities. Furthermore, factors such as limited training dataset size, inconsistent annotation standards, and bias in external validation cohorts mean that most tools remain in the experimental stage and have not undergone prospective validation; therefore, they are not yet suitable for routine clinical application. Since PET images inherently have poor contrast and cannot display fine anatomical structures, it is necessary to integrate multimodal data (including clinical, pathological, and genomic information) and develop interpretable AI technologies to enhance model transparency and clinical credibility when conducting large-scale prospective studies.

**Table 2:** Objectives and key outcomes of AI models for PSMA-diagnosed prostate cancer.

Study(author, year)	AI model and research objectives	Main finding	Data
Karpinski,2024 (40)	ML(Cox,LASSO) The first large-scale, multicenter evaluation of the prognostic value of PSMA-PET combined with PROMISE criteria. A validated whole-stage prostate cancer overall survival prediction model was developed based on PSMA-PET imaging features and compared with traditional scoring systems.	• The C-index for quantitative and visual nomograms was 0.80, 0.78, 0.77, and 0.77 in the internal and external validation cohorts. • Among patients with initial staging, the quantitative nomogram outperformed the STARCAP risk score with AUC values of 0.73 vs. 0.54. • Patients with BCR, both quantitative and visual columnar histogram approaches outperformed the EAU risk score (quantitative: AUC 0.69 vs. 0.52; visual: AUC 0.64 vs. 0.52). • In all-stage patients, both quantitative and visual nomograms outperformed the NCCN risk score (quantitative: AUC 0.81 vs. 0.74; visual: AUC 0.79 vs. 0.73).	Training Set:1110 Internal Test Set:502 External Test Set:802
Kersting,2025 (34)	DL(GAN) Reducing the duration of a single PSMA-PET scan to just a few minutes, further lowering the injected activity, minimizing patient radiation exposure, and reducing motion artifacts achieves a balance between efficiency and value.	• AI-enhanced synthetic PET images exhibit significantly reduced noise compared to original ultrarapid images, achieving visual quality closer to standard PET imaging. • AI-enhanced images substantially improve lesion detection rates across most anatomical regions. Bone metastasis detection rates increased from 72.1% to 85.7%, while overall lesion detection rates rose by 17.9% across all regions.	Training Set:286 Testing Set:71
Kendrick,2023 (41)	DL(U-Net) This study validates the prognostic value of the RECIP 1.0 scoring system in patients with hemicBCR of prostate cancer for the first time, innovatively integrating AI-driven automated segmentation technology to minimize manual intervention.	• Using manual semiautomated segmentation, 13.1% were classified as having RECIP disease progression. These patients had a 3.78-fold higher risk of death compared to nonprogressive patients, with significantly shorter overall survival. • Using AI-based fully automated segmentation, 16.2% were classified as having RECIP disease progression. These patients similarly had a 3.75-fold higher risk of death compared to nonprogressive patients, with significantly shorter overall survival. • Tumor volumes calculated by the AI model showed high correlation with manual measurements (AUC > 0.88).	Training Set:125 Testing Set:74
Marturano,2023 (42)	ML(LOSSO) Develop a predictive model integrating radiomic features and clinical parameters to help identify patients at high risk of BCR prior to treatment.	• Using only clinical data (PSA, Gleason score, clinical stage), the median area AUC for predicting BCR was 0.73. • Performance improved when clinical data were combined with radiomic features. The optimal model achieved a median AUC of 0.78 on the test set. • Clinical parameters—particularly Gleason score and PSA—were the most frequently selected predictors in the models.	Training Set:55 Testing Set:19
Trägårdh,2025 (43)	DL(U-Net) Develop and validate a fully automated AI system that surpasses the performance of previous models in detecting and quantifying prostate cancer lesions in PET-CT images.	• Prostate tumors/recurrence: Sensitivity 84.9%, PPV 85.0%. False-positive lesions decreased from an average of 0.58 per patient to 0.14. • Lymph node metastasis: Sensitivity 90.7%, PPV 63.0%. False-positive lesions decreased from an average of 2.85 per patient to 1.08. • Bone Metastasis: Sensitivity 60.6%, PPV 69.2%. False-positive lesions decreased from an average of 3.20 per patient to 0.76.	Training Set:840 Testing Set:240
Korb,2025 (44)	DL(CNN) Develop a high-precision, fully automated AI model for detecting local recurrence of prostate cancer in PET/CT images.	• After a series of optimizations, the validation set accuracy improved from 56.4% in Model A to 77.1% in the best model (Model D). • On an independent test set, the best-performing Model D achieved an accuracy of 71.0%, specificity of 88.8%, and low sensitivity of 56.8%. • Focusing on the periprostatic region (Model B) increased accuracy by 14.3% compared to whole-body imaging (Model A), with further performance gains achieved by incorporating clinical data (Model C).	Training Set:1016 Testing Set:200
Hein,2025 (45)	DL(CNN) Develop a fully automated AI model for automatic multilesion matching and tracking in continuous PSMA PET/CT scans.	• The optimal model (2D CNN + CT image blocks) achieved an accuracy of 83% and AUC = 0.91 on the test set. • For the task of matching the same lesion, the accuracy reached 89%, approaching the level of physicians.	Training Set:25 Testing Set:5
Benitez,2025 (46)	DL(CNN) Evaluate the performance of the AI-automated PROMISE platform in assessing treatment response in metastatic prostate cancer using PSMA PET/CT after integrating the PROMISE V2 and RECIP 1.0 standards.	• The PROMISE platform achieved 89.6% agreement with nuclear medicine physicians' assessments when classifying patient treatment responses. • The platform demonstrated excellent performance in detecting new lesions, with 89.6% agreement with physician judgments, 80% sensitivity, and 100% specificity.	Testing Set:67
Zang,2025 (47)	DL(U-Net) Evaluate the predictive value of AI-driven fully automated PSMA PET tumor burden quantification for overall survival in patients with metastatic castration-resistant prostate cancer (mCRPC) undergoing 177Lu-PSMA therapy, and develop an imaging-clinical integrated prognostic tool.	• The vast majority of automated segmentation results were rated as “acceptable” or requiring only “minor modifications.” Intra- and interrater agreement was excellent (ICC > 0.7). • Univariate analysis demonstrated that higher AI-quantified tumor burden parameters (PSMATV, PSMATU, PSMATQ) were significantly associated with shorter overall survival (OS). PSMATQ (hazard ratio HR 1.18) emerged as an independent predictor of OS. • The prognostic nomogram (incorporating PSMATQ and clinical factors) achieved a C-index of 0.71 and effectively stratified risk. Median OS in the low-risk group was significantly superior to that in the high-risk group (30.9 vs. 7.9 months).	Training Set:1293 Testing Set:150
Zhao,2025 (48)	DL(CNN) An AI framework based on PSMA PET/CT can automatically evaluate prostate cancer lesions and predict treatment response and risk, supporting clinical decision-making and precision management.	• In the internal validation set, the model demonstrated high accuracy for PSMA-RADS scoring and benign/malignant classification, achieving AUC values of 0.81 and 0.79, respectively. In the prospective validation set, it maintained AUC values of 0.72 and 0.76. • For the most challenging PSMA-RADS-3 lesions, the AI model correctly predicted 82% of benign lesions and 71% of malignant lesions. • In predicting treatment response, the model achieved AUC values of 0.74 and 0.70 in the internal validation and prospective validation cohorts, respectively. In predicting patient survival, the model achieved C-statistics of 0.58 and 0.60 in the two cohorts, respectively.	Training Set:238 Testing Set:36
Huang, 2024 (49)	DL(3D U-Net) Developed a fully automated system for automatic segmentation of metastatic lesions in whole-body PSMA PET/CT scans for recurrent prostate cancer.	• The AI-detected regions showed high overlap with the “gold standard” annotations by physicians (Dice coefficient: 0.63 internally, 0.60 externally). • The model demonstrated high precision, with over 88% of detected lesions being true positives. The AI-calculated whole-body tumor burden metrics highly correlated with physician manual calculations (R² > 0.99).	Training Set:97 Testing Set:19
Trägårdh,2022 (50)	DL(3D U-Net) Develop and open-source AI tools to enable automated objective detection of pelvic lymph node metastases in PSMA PET-CT scans, facilitating global research validation collaboration.	• The AI model demonstrated an average sensitivity of 82%, surpassing the 77% achieved when three physicians compared their findings. • The AI model generated an average of 1.8 false-positive lesions per patient. This number is significantly lower than those reported in other similar studies, indicating its excellent specificity.	Training Set:161 Testing Set:50
Capobianco,2022 (51)	DL(CNN) Develop an AI system to automatically analyze PSMA PET/CT whole-body scans of prostate cancer patients, identify lesions and distinguish physiological uptake, automatically localize them, and provide N and M staging.	• On the test set, the model achieved an average precision of 80.4% and a sensitivity of 81.1% in identifying suspicious lesions. • The model's agreement rates with expert assessments for regional lymph node metastasis and distant metastasis detection were 81% and 77%, respectively. • Experiments demonstrated that incorporating ¹^8^F-FDG data significantly improved model performance on ^6^^8^Ga-PSMA-11 data—particularly in anatomical location classification—regardless of whether transfer learning or ensemble training was employed.	Training Set:121 Testing Set:52
Jafari,2024 (52)	DL(nnU-Net) Develop a fully automated DL-based tool to delineate metastatic lesions throughout the body in prostate cancer. This aims to enhance efficiency, reduce human error, and support personalized medicine.	• On the internal test set, the model achieved an accuracy of 83% and a sensitivity of 92% in identifying whether patients had tumor lesions. • Across all test datasets, the accuracy for detecting individual lesions ranged from 87% to 94%, and the sensitivity ranged from 88% to 95%. • When comparing the boundaries outlined by the model to those manually drawn by physicians, the core metric DSC (Discrete Simultaneous Consistency) averaged 70% on the internal test set.	Training Set:200 Internal Test Set:133 External Test Set1:47 External Test Set2:32

### 3.3 AI+biopsy and pathological techniques for improved prostate cancer diagnosis and gleason grading accuracy

Transrectal ultrasound (TRUS)-guided core needle biopsy, the gold standard for prostate cancer diagnosis, was originally employed with a 12-core sampling technique targeting common tumor locations in the prostate. Since the needles do not directly penetrate the tumor tissue, an increased rate of missed diagnoses has been led to by this approach. Diagnosis relies entirely on tissue sections being examined by pathologists under optical microscopes, with grading performed via the Gleason scoring system. Significant variability among observers has been caused by this method—scores for the same specimen can differ by 1–3 points between pathologists (53).

As the incidence of cancer continues to rise, the workload of pathologists has intensified. The shift from traditional stained slides to WSI, which provides comprehensive information, has enabled diagnosis to be conducted independently of spatial and temporal constraints (54). While digital pathology eliminates the need for optical microscopy and allows high-resolution images to be stored and shared, it has not resolved issues related to diagnostic efficiency and standardization. With the rapid advancements in precision medicine and AI, targeted biopsies have now been enabled to reduce missed diagnoses, enhance differentiation between benign and malignant lesions, and improve diagnostic accuracy.

AI-guided targeted biopsies (RA-TB) using MP-MRI-TRUS image fusion were pioneered by Kaufmann (55), and the results revealed that 85% of biopsy-confirmed cancers were detected exclusively via RA-TB. AI-assisted targeted needle biopsy based on MP-MRI-TRUS fusion images was employed by Lee,et al (56), and a 46% detection rate for CS-PCa was achieved among 433 patients who underwent both targeted and systematic biopsies. The precise localization of biopsy sites is enabled by AI-guided targeted biopsy, and subsequent histopathological analysis is aided by accurate sampling.

The pathological diagnosis of prostate cancer has been revolutionized by AI. An AI algorithm was developed by Steiner,et al. (57), through which the diagnostic accuracy for prostate cancer was elevated to 95.8%, while the average observation time was reduced. The Paige prostate AI model was proposed by Perincheri,et al. (58) To classify WSI as suspicious or nonsuspicious, a positive predictive value of 97.9%, a negative predictive value of 99.2%, a sensitivity of 97.7%, and a specificity of 99.3% were achieved. DL plays a significant role in pathological image analysis. A deep neural network (DNN)-based AI model was employed by Ström (59) to process section images from prostate biopsy samples. Exceptional accuracy was demonstrated by this model in distinguishing benign from malignant biopsies, with an AUC value close to 1 (AUC = 0.997 on the internal test set and AUC = 0.986 on the external validation set); additionally, accuracy comparable to that of pathologists was achieved in terms of Gleason grading.

An AI-based algorithm for precise prostate cancer diagnosis was developed by Pantanowitz (60), and further improvements in cancer detection performance were achieved (AUC = 0.997 on the internal training set and AUC = 0.991 on the external validation set). Strong performance was also demonstrated by the algorithm in distinguishing high-grade from low-grade cancers on the basis of Gleason scores (AUC = 0.941). These algorithms exhibit considerable accuracy in identifying prostate cancer and determining its grade, with good consistency shown between their results and those of uropathologists—thus validating their feasibility in the pathological diagnosis of prostate cancer.

The PANDA challenge, aimed at developing and optimizing AI algorithms to increase the accuracy and reproducibility of prostate cancer diagnosis and Gleason grading, achieved an optimal algorithm performance of 0.931 on the internal validation set; moreover, high consistency with uropathologists was achieved on external validation sets from different countries, and the rate of missed diagnoses was reduced simultaneously (61). Traditional pathology sections require extensive staining time, so reducing the staining duration is regarded as a significant advancement for pathology departments. An AI convolutional neural network was employed by Mannas et al. (62) to analyze pseudocolor-processed stained images generated by stimulated Raman spectroscopy (SRS) microscopy. This technique utilizes Raman light reflected at different wavelengths to produce images with distinct colors. Prostate biopsy images were generated via AI-driven SRS within 2–2.75 minutes, and cancerous prostate biopsies were classified within approximately 1 minute. For prostate cancer detection, an accuracy of 96.5%, a sensitivity of 96.3%, and a specificity of 96.6% were achieved by AI.

The (**Table 3**) summaries the main applications of AI in pathological imaging over the past five years. For fine-needle aspiration biopsies and WSI following radical surgery, AI automatically identifies areas of normal glandular tissue, benign hyperplasia and cancerous tissue. These represent the most fundamental functions of AI in prostate cancer pathology. Through pre-processing, 70–80% of invalid image regions can be filtered out, enabling the detection of occult lesions missed during the pathologist’s initial diagnosis, thereby reducing reading time whilst increasing detection rates. The core applications lie in AI’s specific recognition of tumor glandular morphology, the completion of Gleason scoring and ISUP grading in accordance with international standards, and the provision of quantitative evidence for risk stratification. However, limitations are inevitable. In the grey areas between Gleason 3 + 4 and 4 + 3, and for PI-RADS=3, AI performance declines significantly and fails to match expert levels. Some benign hyperplasia and inflammatory tissue are easily misclassified as tumors by the AI; detection rates for low-incidence high-risk features, such as lymphovascular invasion and perineural invasion, remain low. AI focuses primarily on morphology and has limited capacity for in-depth analysis of genetic abnormalities and protein expression, making it difficult to integrate genomic data for predictive purposes. Currently, research remains predominantly single-centre and retrospective; the annotation of whole-slide images is labor-intensive and requires a high level of expertise, whilst cross-institutional data sharing is constrained by privacy and legal restrictions, resulting in a scarcity of large-scale, multi-centre prospective randomized controlled trials. There remains a need to advance the development of standardized, multi-centre datasets, optimize models’ robustness and interpretability, and conduct prospective clinical studies to strengthen findings; simultaneously, exploration of cutting-edge areas such as multimodal fusion (81), virtual staining and pathogenomics should be pursued.

**Table 3:** Fully automated pathology AI model.

Study(author, year)	AI model and objectives	Main finding	Data
Xie,2022 (63)	DL(GAN) A fully automated 3D pathological analysis system has been developed that enables non-destructive 3D imaging of biopsy samples and automatically quantifies glandular morphological features. By utilizing intuitive glandular morphological features that are easily understood by pathologists, the system demonstrates that 3D pathology offers significant additional prognostic value.	• A multiparameter model incorporating 12 3D features achieved an AUC (area under the curve) of 0.81 for predicting biochemical recurrence within 5 years. • The 3D feature-based model effectively stratified patients into high-risk and low-risk groups, with a hazard ratio (HR) of 11.2 indicating that the high-risk group faced an 11-fold higher recurrence risk than the low-risk group, demonstrating exceptional risk stratification capability.	Training Set:50 Testing Set:50
Flannery,2025 (64)	DL(CNN) Studies have shown that there are systematic morphological differences between prostate biopsy specimens and surgical specimens, and artificial intelligence detection models designed for these two types of specimens cannot be universally applied. In clinical practice, it is essential to use models tailored to specific sample types; otherwise, classification errors may occur, which could in turn affect treatment decisions.	• The model's performance plummeted on unfamiliar sample types. For instance, the biopsy model's F1 score for surgical specimens dropped from 0.93 to 0.64. • Visualization revealed that the biopsy model was accustomed to examining “compressed” glands, while the surgical model was accustomed to examining “intact” glands.	Training Set:150 External Test Set1:100 External Test Set2:350
Salvi,2023 (65)	DL(K-PPM) To develop a fully automated AI system capable of automatically analyzing WSIs of prostate biopsy specimens stained using immunohistochemistry (IHC), accurately segmenting different types of glandular tissue (benign, PIN, adenocarcinoma), thereby providing pathologists with an objective, quantitative decision-support tool.	• On the test set, the model DSC = 90.36 and an average balanced accuracy (BalACC) of 94.24%. This indicates that the model's segmentation results highly align with pathologists' manual annotations. • The highest segmentation accuracy was observed for adenocarcinoma and benign glands. For the most challenging PIN, the model still attained a Dice coefficient of 79.8%. • At the whole-slide level, the average absolute error between the model's automatically calculated glandular areas and manual measurements was only 1.64%.	Training Set:27 Testing Set:5
Song,2024 (66)	DL(CNN) To develope the TriPath computational platform to address the sampling bias inherent in traditional two-dimensional pathology due to limited tissue sampling. By processing vast amounts of three-dimensional pathological image data and employing weakly supervised learning using only patient-level labels, we validated the value and potential of three-dimensional pathology in clinical prognosis assessment.	• 3D whole-volume analysis (AUC = 0.86) > 2D (AUC = 0.82) > baseline clinical Gleason score (AUC = 0.76). • Model prognostic accuracy surpasses evaluations by six senior pathologists on the same 3D dataset • Analyzing the entire tissue volume significantly reduces risk prediction variability caused by random sampling, yielding more reliable results. AI-identified high-risk areas correlate with known poor prognostic histologies, providing interpretable outcomes.	Training Set:50 Testing Set:24
Huang,2022 (67)	DL(CNN) Proposing a classification network model that integrates seven manually designed features with a deep learning feature for cancer detection in prostate pathology images. We evaluate whether this integrated model can more effectively focus on key pathology-relevant regions, thereby improving clinical reliability.	• The ensemble model centered on ResNet-18 achieved an accuracy of 95.45% on the test set, with an AUC as high as 0.9834, demonstrating balanced sensitivity and specificity. • The ensemble model's performance significantly outperformed any single feature (all single features had accuracy <90%), proving the success of the ensemble strategy.	Training Set:880 Testing Set:220
Erak,2023 (68)	DL(Transformer) Developing a DL model capable of predicting two key molecular subtypes of prostate cancer solely by analyzing images of routine hematoxylin-and-eosin (H&E)-stained histopathological sections. This is a major highlight of the study. The PTEN loss heatmap generated by the AI showed a high degree of consistency with the loss regions annotated by pathologists based on PTEN immunohistochemical staining.	• ERG predictive performance: In the RP validation cohort, AUC = 0.86-0.91, while in the more challenging needle biopsy cohort, AUC still reached 0.78-0.80. • PTEN predictive performance: In the RP validation cohort, AUC = 0.72-0.81, while in the needle biopsy cohort, AUC = 0.75.	Training Set:429 Testing Set:1772
Wong,2024 (69)	DL(GAN) Demonstrate that images generated by virtual staining—a generative AI technique—are diagnostically equivalent to traditional chemical staining methods, and establish rigorous evaluation standards for this approach.	• The validated Gleason grading AI model, trained on authentic H&E images, demonstrated exceptionally high scoring consistency on virtual H&E images (weighted Kappa=0.902). • The accuracy of pathologists performing Gleason grading using virtual H&E images achieved statistical noninferiority compared to authentic H&E images.	Training Set:796
Kott,2021 (70)	DL(ResNet) Develop and validate a deep learning algorithm for the primary diagnosis (benign/malignant determination) and Gleason grading of prostate cancer biopsies.	• The model achieved 91.5% accuracy in distinguishing benign from malignant tissue, with a sensitivity of 93% and specificity of 90% for identifying cancerous tissue. • The model achieved 85.4% accuracy in the Gleason grading task.	Training Set:25
Ding,2024 (71)	DL(AIRAProstate) Evaluate whether AI grading algorithms can predict long-term clinical outcomes in actively monitored patients (grade reclassification) beyond merely mimicking pathologist scoring, with results directly applicable to optimizing active surveillance strategies for more precise personalized treatment.	• AIRAProstate achieves 95%-97% agreement with urological pathologists in cancer diagnosis and 72%-74% agreement in precise GG grading. • For patients whose AI upgrade was followed by surgery, 97% (28/29) confirmed Gleason pattern ≥4 in their surgical specimens (indicating the cancer was indeed more severe), demonstrating the biological validity of AI predictions.	Training Set:138 Testing Set:169
Xiang, 2023 (72)	DL(CNN) Develop an AI system that requires only whole-slide-level labeling (weakly supervised) to automatically diagnose and grade prostate cancer, addressing the high annotation costs and interobserver variability inherent in traditional methods	• On two external validation sets, the model achieved AUC values of 0.985 and 0.986, respectively, for distinguishing between cancerous and benign tissue, with sensitivity exceeding 99% in both cases. • On the internal validation set, the model showed high agreement with the pathologist’s gold standard, with a weighted Kappa coefficient of 0.931. On external validation set 1, the coefficient was 0.801.	Training Set:10616 Internal Test Set:531 External Test Set1:844 External Test Set2:4675
Serafin,2023 (73)	DL(CELLPOSE),LASSO This is a systematic study examining the prognostic value of 3D whole-cell nuclear morphology in human prostate cancer tissue. Using three-dimensional (3D) microscopy, intact prostate biopsy specimens are imaged non-invasively. Combined with artificial intelligence analysis of three-dimensional nuclear morphological features, this method can more accurately predict the aggressiveness of the cancer.	• In predicting hemicBCR within 5 years, the model based on 3D nuclear morphology features significantly outperformed the model based on 2D features (AUC = 0.75 VS 0.62). • The study revealed that invasive cancer cells exhibit more regular, near-spherical three-dimensional nuclei and exhibit reduced morphological heterogeneity compared to noninvasive cells. • After stratifying patients into high-risk and low-risk groups using the 3D nuclear feature model, significant differences in biochemical recurrence-free survival were observed between the two groups.	Training Set:46
Wessels,2021 (74)	DL(CNN) Using AI technology to predict whether cancer cells have metastasized to pelvic lymph nodes by analyzing routine hematoxylin-and-eosin (H&E) stained histopathological sections of primary prostate tumors.	• On an independent test set, the AI model achieved an overall performance AUC=0.68 in predicting lymph node metastasis. • The risk score predicted by the AI model and lymphatic vessel invasion (LVI) serve as two independent predictors for lymph node metastasis, elevating predictive performance to 0.83.	Training Set:118 Testing Set:100
Koivukoski, 2023 (75)	DL(GAN)	• 1.5-micron-thick tissue sections achieve optimal results for virtual staining when deparaffinized without mounting. • Virtual H&E images generated using the pix2pix model (supervised learning) exhibit significantly higher fidelity in reproducing cellular details such as nuclei and cytoplasm compared to the CycleGAN model, even revealing difficult-to-identify basal cells.	
Pinckaers, 2021 (76)	DL(CNN) Develop a method that directly utilizes WSIs and simple clinical diagnostic labels—without the need for pixel-level annotation—to achieve advanced prostate cancer detection performance, thereby providing a more practical training approach for AI in pathology.	• On the internal test set, the model AUC = 0.992, comparable to advanced MIL methods (AUC = 0.990). • On the external test set (Olympus dataset), AUC=0.909 significantly outperformed the MIL model (AUC = 0.799). • When trained using only 5% of the training data (250 samples), the model (0.971) still outperformed the MIL model (0.965), demonstrating its higher data utilization efficiency.	Training Set:4712 Internal Test Set:535 External Test Set:205
Cha,2025 (77)	DL Develop and validate an AI algorithm based solely on histopathology (H&E-stained slides) to predict the risk of metastasis in prostate cancer patients following radical prostatectomy (RP).	• The performance of the WSI AI model in the test cohort (C-index: 0.81–0.85) was comparable to genomic classifiers such as Decipher (0.72) and Polaris (0.80). • The TMA AI model demonstrated robust predictive capability (C-index: 0.71) in a large external validation cohort (HPFS/PHS) using only approximately 1 mm² of tissue. • When combined with CAPRA scores or pathological grading, the AI risk score significantly outperformed any single tool in predictive performance (C-index: 0.83–0.95).	WSI AI model:303 TMA AI model:1351
Pizurica,2023 (78)	DL(ResNet-18) Develop and validate a deep learning model named TiDo that can predict the presence of TP53 gene mutations using only WSIs of H&E-stained prostate cancer tissue.	• On the TCGA test set, the optimal model predicted TP53 mutation with AUC = 0.71. In the independent UZhent cohort, the model showed strong generalizability at the patient level and at the lesion level (AUC = 0.65 for the primary lesions). • G expression and cell-type analyses revealed that the TiDo model relied on tumor cell-specific and tumor microenvironment-specific (specifically cancer-associated fibroblasts, Cs) features when making predictions. • Even in patients without a TP53 mutation, the model’s high predictive score was significantly associated with disease aggressiveness metrics such as lymph metastases and biochemical recurrence.	Training Set:365 Testing Set:41
Ikromjanov,2023 (79)	DL(U-Net) Develop a high-performance automated segmentation system (Auto Annotation System, AAS) capable of accurately distinguishing cancerous regions, benign glandular regions, and stroma regions within WSIs.	• On the test set, the model proposed in this paper achieved an average Dice coefficient (ADC) of 0.891. In comparisons across various metrics, this model outperforms all other pre-trained models. For example, its average Dice coefficient is 8.9% higher than that of the baseline model ResNet34-U-Net and 1.4% higher than that of the single-model EfficientNetB2-U-Net.Λ	Training Set:8100 Testing Set:900
Zabihollahy,2025 (80)	DL(U-Net+Transformer) Develop a fully automated, explainable AI tool for automatically detecting lymph node metastasis (LNM) in prostate cancer patients' histopathological slides to assist pathologists in their work	• On the test set, the model AUC = 0.94, with a sensitivity of 96% and specificity of 92%. • The AI model successfully detected 17 cases of micrometastases missed by pathologists. In the internal dataset, the pathologist miss rate was 9%, while the AI model's miss rate was only 3%. • The AI model analyzes a single slide containing 1–5 lymphoid tissues in an average of just 230 seconds, significantly faster than the examination times of experienced pathologists.	Training Set:345 Internal Test Set:545 External Test Set1:140 External Test Set2:102

### 3.4 AI-based prediction of invasive risks in prostate cancer

Significant progress has been made in the application of AI technologies to predict extracapsular extension (ECE) and seminal vesicle invasion (SVI) in prostate cancer. Predictive accuracy is enhanced by AI models through the integration of imaging and clinical data, thereby supporting clinical decision-making. High efficiency in the automated segmentation of the ECE and SVI is demonstrated by deep learning models, particularly CNNs. AI-based models developed for ECE detection improve predictive accuracy by analyzing MR images to segment lesions (82).

In one study, automated segmentation and prediction of ECE were achieved via a deep learning model based on MP-MRI and PSMA PET/CT data, with robust performance demonstrated via cross-center validation (83).Despite the improvements in the efficiency of these applications, challenges such as overfitting in small sample sizes and limitations in segmentation accuracy are faced. A prospective matched cohort study revealed that the sensitivity of PSMA PET for ECE detection was 75%, which was significantly greater than the 63% achieved by MP-MRI (P = 0.01). However, no statistically significant difference was found in the sensitivity for SVI detection between the two methods (91% vs. 85%, P = 0.07) (84, 85).

With respect to the accuracy of local tumor staging, a significantly AUC was demonstrated by PSMA PET than by MP-MRI (45% vs. 28%) (86) Furthermore, compared with both MP-MRI (38.9% sensitivity, 82.6% specificity) and CT (38.5% sensitivity, 83.6% specificity), PSMA PET has 73.7% sensitivity and 97.5% specificity for detecting lymph node metastasis (87).

A multicenter retrospective study further confirmed, through multivariate analysis, the concordance between MRI-detected ECE (including primary capsule contact, extracapsular invasion, seminal vesicle invasion, or organ invasion) and postoperative pathology. The central role of pathological validation in model optimization was demonstrated in this study (88). These findings indicate that models trained on single-institution data have limited generalizability to cohorts from other medical centers. During external validation, the predictive model developed by the Michigan Urological Surgery Improvement Consortium (MUSIC) showed differing predictive performance for ECE, SVI, and lymph node invasion (LNI) compared with the MSK model. The clinical characteristics of black patients, such as higher prostate-specific antigen levels, may further affect the universality of the models. The study also included 388 patients from three medical centers, exploring the potential of cross-center data to enhance model generalizability through a deep learning model that integrates MP- MRI and PSMA PET (89).

Recent studies (**Table 4**) have demonstrated that preoperative non-invasive prediction of the risk, extent, and grade of ECE and SVI based on MP-MRI and PSMA PET imaging holds great potential to replace conventional invasive biopsy, enabling precise preoperative risk stratification for prostate cancer patients. WSI-assisted automated grading and evaluation of adverse pathological features in postoperative prostate specimens can provide robust evidence for clinical decision-making, facilitating the selection of appropriate adjuvant therapies and the development of personalized intervention strategies. Despite these promising advances, further large-scale, multicenter prospective studies are still warranted to mitigate the impacts of data heterogeneity and improve the generalizability and robustness of predictive models across diverse patient populations. Additionally, the poor interpretability of AI features remains a critical challenge. The biological associations between AI-derived imaging biomarkers (e.g., MRI signal heterogeneity and PET tracer uptake patterns) and pathological outcomes have not been fully clarified, rendering the decision-making process of AI models a “black box” (99). Such limited interpretability considerably undermines clinicians’ confidence in AI-based predictive results, especially when model predictions are inconsistent with conventional clinical experience.

**Table 4:** Adverse pathology prediction models for recent years.

Study(author, year)	AI model and objectives	Main finding	Data
van den Berg,2023 (90)	RF,ET,LR For the first time at the lesion-specific level, this study combined traditional MRI radiomic features (such as tumor contact length, TCL) with innovative three-dimensional geometric features (such as tumor contact surface area, TCSA).	• All three prediction models are demonstrated excellent performance at the lesion-specific level with AUC = 0.86-0.91. • On the external test set, the RF model achieved significantly higher accuracy (83% vs. 67%) and specificity (89% vs. 69%) compared to radiologists.	Training Set:524 Internal Test Set:162 External Test Set:189
Simon, 2024 (91)	RF Develop a fully automated AI model that requires no human intervention, from image input to result output, and makes judgments based on the anatomical structure of the prostate and the geometric relationships of lesions, ensuring that the results are highly interpretable.	• On the test set, the best model achieved a balanced accuracy of 0.390 ± 0.078; its AUC values for EPE grades 0–3 were 0.70, 0.65, 0.68, and 0.55, respectively. • The model demonstrated a sensitivity of 0.67, specificity of 0.73, and accuracy of 0.72, comparable to radiologists' performance.	Training Set:507 Internal Test Set:127
Xu,2020 (92)	LASSO The study validated an objective assessment tool that does not rely on the subjective experience of radiologists. This tool utilizes all standard sequences in MP-MRI of the prostate to perform an imageomics analysis of EPE, thereby addressing the issue of low sensitivity in EPE detection associated with subjective MRI assessments at the time.	• On the validation set, the AUC was 0.865, with an accuracy of 81.8%, sensitivity of 71.4%, and specificity of 89.5%. • The performance of the radiomics model was significantly superior to that of the model using clinical variables alone (AUC 0.865 vs. 0.658).	Training Set:82 Internal Test Set:33
Liu, 2025 (93)	DL(Transformer) By applying the TabPFN base model to the field of radiomics, we leveraged its learning capabilities to overcome the reliance of traditional models on massive datasets and complex parameter tuning. The model demonstrated robust performance in external validation across multiple centers and imaging modalities, addressing the key limitation of poor generalization in traditional radiomics models.	• The model demonstrated superior performance on both internal and external test sets, achieving AUC of 0.806 and 0.842, respectively; training time was significantly reduced to 1.2 seconds. • The AI-enhanced algorithm markedly improves diagnostic accuracy for less experienced radiologists and substantially increases interobserver agreement (with Kappa values reaching up to 0.98).	Training Set:287 Internal Test Set:124 External Test Set:102
Yao,2025 (94)	DL developed a ResNet-50-based multimodal DL model that integrates MP-MRII and PSMA-PET/CT information to predict ECE in prostate cancer with high accuracy. Its performance surpasses that of single-modality imaging models and significantly enhances diagnostic accuracy for radiologists.	• On the test set at Centre 1, the MP-MRI+PET/CT multimodal model achieved an AUC of 0.821, whereas the standalone MP-MRI and PET/CT models yielded AUC values of 0.76 and 0.77, respectively. • At Centre 1, physician scores improved significantly from an AUC of 0.64 to 0.82 following AI augmentation. At Centre 2, scores similarly rose from 0.68 to 0.87. This demonstrates that AI effectively compensates for limitations in physician subjective assessment.	Training Set:177 Internal Test Set:28 External Test Set:36
Zhao,2024 (95)	DL(3D Swin-Transformer) Develop and validate a deep learning model based on the 3D Swin-Transformer architecture, utilizing preoperative MP-MRI images to predict the presence of AP in prostate cancer patients postsurgery. Concurrently, construct an ensemble model (TransCL) integrating imaging and clinical features to further enhance performance.	• The TransCL integrated model achieved an AUC of over 0.813 for predicting any AP and an AUC as high as 0.916 for predicting SVI. • TransCL’s performance significantly outperformed that of clinical models and radiologists (AUC = 0.749 and 0.664, respectively). • The TransCL model proposed in this study makes its decision-making process interpretable. Clinicians can input specific patient metrics to obtain personalized AP risk probabilities.	Test Set:508 External Test Set:108
Yu, 2025 (96)	DL(Transformer) • Develop a model capable of predicting whether cancer cells EPE solely by analyzing routine preoperative prostate biopsy slides. This will enable clinicians to more accurately assess the aggressiveness of the cancer prior to surgery and develop more personalized surgical plans.	• The model achieved an AUC of 0.886 for predicting EPE on an independent test set, with an accuracy of 86.4%. • The regions targeted by the model exhibit distinct malignant biological characteristics (such as marked nuclear atypia and infiltrative growth), aligning with pathological knowledge. • Patients predicted by the model to be at high EPE risk indeed exhibited a higher risk of postoperative hemicBCR.	Training Set:177 Testing Set:44
Moroianu,2022 (97)	DL(U-Net) combines two existing pre-trained deep learning models to detect and localize EPE of prostate cancer in MP-MRI.	• On the independent test set, EPENet demonstrated a sensitivity of 80.0% for detecting EPE, whereas radiologists achieved only 50.0%. • When dividing the prostate into six regions corresponding to needle biopsy sites, EPENet achieved a sensitivity of 61.1% and a specificity of 58.3% at the six-segment level..	Training Set:74 Testing Set:49
Pinckaers,2022 (98)	DL(CNN) Investigating whether deep learning can surpass traditional ISUP grading by automatically identifying more subtle, prognostically significant features across the entire morphology of prostate cancer tissue, thereby more accurately predicting the risk of BCR following radical prostatectomy.	• AI biomarkers are significantly associated with biochemical recurrence; for every 1-point increase in the model’s predictive score, the risk of recurrence increases approximately threefold (OR = 3.32) • The model demonstrates enhanced predictive power, with a HR as high as 5.78. In multivariate analysis, its predictive power remains highly statistically significant (HR = 3.02).	Training Set:685 Testing Set:204

### 3.5 AI + multimodal data for further improve precision diagnosis and prognostic evaluation of prostate cancer

**Figure 3:**
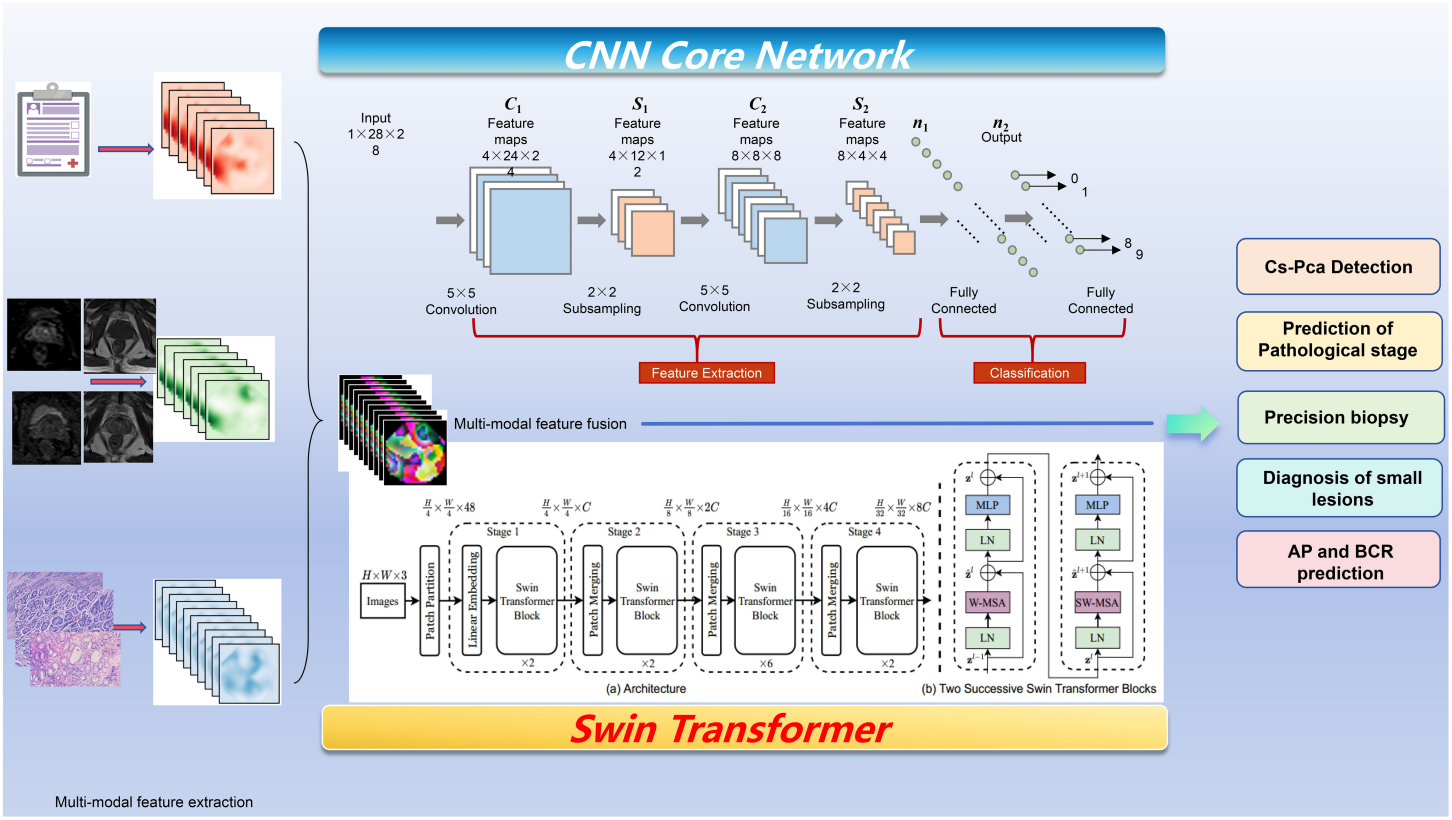
The internal architecture used for multi-modal diagnosis of prostate cancer with CNN and transformer structures, Created with MedPeer (medpeer.cn).

Significant challenges are faced in the diagnosis and prognostic assessment of prostate cancer due to tumor differences. A comprehensive reflection of the biological characteristics of tumors is difficult for single-type data. A more complete understanding of tumor biology is provided by integrating multidimensional information from imaging, molecular indicators, and histopathology *(***Figure 3***)*. This integration holds important biological and clinical significance for achieving accurate risk classification and prognostic evaluation (100). Not only are the limitations of single data types overcome by this multimodal fusion strategy, but deeper insights into tumor differences, invasive potential, and disease progression paths are also enabled through the combination of complementary information. These findings lay the foundation for the development of personalized treatment strategies.

MP-MRI, which combines T2WI, diffusion-weighted imaging (DWI), and dynamic contrast-enhanced (DCE) sequences, provides anatomical structure, tissue cell density, and blood flow information of the prostate simultaneously. Its value in diagnosing clinically significant prostate cancer has been confirmed by numerous clinical studies. PSMA PET/CT achieves highly sensitive tumor imaging by targeting PSMA molecular expression, showing significant advantages in detecting metastatic lesions (especially bone metastases) (101). However, relatively low sensitivity is still exhibited by this method for low-grade tumors with weak PSMA expression (102).

Histopathology, regarded as the gold standard for prostate cancer diagnosis, provides microscopic structural characteristics (such as Gleason grading). Digitized WSI support AI-driven quantitative analysis—for example, optimization of International Society of Urological Pathology (ISUP) grading is achieved through the correlation of histological features with imaging-related data (103). Cross-modal fusion is the core technology of multimodal AI and can be divided into early-stage fusion (feature-level) and late-stage fusion (decision-level). During the raw feature extraction phase, multisource data (such as MR images and clinical parameters) are integrated via early-stage fusion, either through direct combination or interactive enhancement of correlations between different data types. For example, in prostate cancer research, multimodal AI classifiers are trained by simultaneously integrating features, including MRI lesion volume, prostate-specific antigen (PSA) levels, prostate volume, and patient age. Complementary information across different data types is fully utilized by this fusion strategy, significantly improving diagnostic performance.

Marked improvements in the accuracy of prostate cancer detection are demonstrated by multimodal AI. Significant advantages have also been shown by multimodal AI technology in predicting the ISUP grade. Research confirms that multimodal models integrating PSMA PET/CT and MP-MRI imaging features perform significantly better than single-type approaches in predicting ISUP staging. This improved accuracy is due mainly to the complementary nature of functional metabolic information and anatomical details (104, 105).

By integrating imaging, pathological and clinical data, multimodal artificial intelligence has demonstrated significant value in the diagnosis and treatment of prostate cancer (**Table 5**). However, the clinical application of current multimodal AI models remains constrained by the availability of high-quality and comprehensive MP-MRI scans, histological slides, genetic sequencing data and long-term follow-up data, which limits the models’ generalization ability. At present, feature concatenation, feature-level fusion and decision-level fusion each have their own advantages and disadvantages, and no universally accepted optimal architecture has yet emerged. There is an urgent need to conduct large-scale, multicentre prospective randomized controlled trials (RCTs) to rigorously validate the diagnostic efficacy and therapeutic guidance value of AI tools in real-world clinical settings. For example, an Asian multicentre study screening for early-stage high-risk prostate cancer using routine health check-up data showed AUC 0.76 in the multi-centre validation cohort; the application of the APCA score reduced unnecessary biopsies by 20.2% and 38.4%. However, in the multi-centre validation cohort, there was a risk of 5.0% and 10.0% of high-risk prostate cancer (HGPCa) cases being missed (115).which will further drive the translation of multimodal AI from cutting-edge research into clinical practice.

**Table 5:** Multimodal AI diagnostic model for prostate cancer diagnosis.

Study (author, year)	AI model and research objectives	Main finding	Data
Bhattacharya,2022 (106)	DL(Correlational Neural Network) Developing the CorrSigNIA model, which integrates MRI and pathological image information to achieve precise detection of prostate cancer and distinguish between indolent and aggressive components, thereby addressing the challenge of MRI's difficulty in differentiating benign tissue, indolent carcinoma, and invasive carcinoma.	• In lesion-level assessment, CorrSigNIA demonstrated superior performance in detecting cancer (AUC: 0.81) and clinically significant cancer (AUC: 0.82–0.86), ranking higher than other comparison models across multiple test cohorts. • • CorrSigNIA achieved 80% accuracy in distinguishing cancer patients from noncancer patients, demonstrating balanced sensitivity and specificity superior to other models. • The model exhibited stable performance across test sets derived from different clinical pathways (surgery vs. biopsy), confirming its strong potential for clinical applicability.	Training Set:98 Testing Set:345
Singh,2023 (107)	DL(EfficientNet-B1) Develop a web application capable of integrating deep learning AI predictions with clinical data (such as biopsy images, electronic health records, etc.), and assess its clinical utility.	• The model demonstrated excellent performance on an external validation set (kappa=0.862). • User evaluation results indicate that 60% of pathologists and medical specialists found the AI prediction results easy to navigate and comprehend. All users strongly favored consolidating all information into a single view, perceiving this as significantly streamlining workflows.	Training Set:12625 Testing Set:940
Tward,2024 (108)	DL(MMAI) Training a mature MMAI model for its prognostic predictive capabilities in a large-scale, multicenter Phase III randomized clinical trial cohort by integrating digital pathology images with clinical data.	• The MMAI risk stratification identified more patients as low risk (43.5% in the MMAI low-risk group versus 30.4% in the NCCN low-risk group) and fewer as high risk (21.8% in the MMAI high-risk group versus 44.1% in the NCCN high-risk group). • Among NCCN high-risk patients, the 10-year metastasis rates for the low-, intermediate-, and high-risk subgroups identified by MMAI were 9.1%, 16.0%, and 26.3%, respectively, demonstrating significant heterogeneity within the NCCN high-risk group.	Training Set:7319 Testing Set:2486
Zhang, 2024 (109)	DL,Logistic Regression Develop a non-invasive AI tool that combines radiomics, clinical data, and deep learning features to predict the presence of perineural invasion (PNI) in prostate cancer. This will help clinicians assess tumor aggressiveness and develop personalized treatment plans.	• The integrated DLRC model achieved AUC values of 0.914 and 0.848 in the training and validation cohorts, respectively, demonstrating significantly superior performance to any single model (clinical, radiomic or deep learning). • The DLRC model demonstrated high concordance between predicted and actual probabilities, with decision curve analysis indicating superior clinical net benefit across a broader threshold range.	Training Set:389 Testing Set:186
Zabihollahy, 2025 (110)	DL(A2-Morph) Develop a rapid, precise AI tool to resolve the spatial alignment challenge between preoperative prostate MRI scans and postoperative histopathological sections (WMHP). This precise alignment forms the cornerstone for providing reliable training data for AI models, validating MRI diagnostic outcomes, and conducting in-depth research into cancer imaging characteristics.	• Model performance metrics for prostate volume:Dice coefficient: 0.95 ± 0.06.Boundary distance (Hausdorff distance): 1.84 mm ± 0.38 • The entire registration workflow (encompassing segmentation, deformation correction, and registration) averages approximately 10 seconds, significantly faster than conventional energy function-optimized approaches.	Training Set:270 Testing Set:45
Rodrigues,2025 (111)	DL(LightGBM) Develop a multimodal fusion AI model that combines radiomics, radiologist assessments (PI-RADS), and patient clinical information (PSA, age, etc.) to more accurately identify CS-PCa.	• On the prospective validation set, the multimodal model achieved an AUC of 0.91, significantly higher than the 0.85 attained by PI-RADS scores. When fixed detection sensitivity matched that of PI-RADS scores (>3 points), the multimodal model demonstrated a specificity of 77%, substantially exceeding PI-RADS's 66%. • SHAP analysis revealed that PI-RADS score and peripheral zone location were the most critical predictors in the model, while radiomics features (particularly first-order and texture features) also contributed significant value.	Training Set:7157 Testing Set:1629
Roest,2024 (81)	DL(nnU-Net) Multimodal artificial intelligence that integrates MRI with clinical parameters through an early fusion strategy has significantly improved the detection accuracy ofCS-PCa, while also identifying key factors influencing the model’s AUC.	• In external validation, multimodal AI demonstrated significantly superior performance compared to unimodal baseline models. • The optimal multimodal AI diagnostic model matched radiologist performance (AUC 0.77:0.75). • Jackknife analysis identified deep learning suspicion scores for primary lesions and PSA levels as the most critical predictors within this model.	Internal Test Set:81 External Test Set:529
Öğülmüş,2025 (112)	DL(3D-CNN) Develop a deep learning model that integrates PET/CT images, radiomic features, and clinical data to make predictions using weakly labeled data for the purpose of predicting lymph node metastasis in patients with intermediate- to high-risk prostate cancer.	• On the test set, the multimodal AI model (imaging informatics + clinical data) achieved an average accuracy of 0.85 ± 0.03 and an F1 score of 0.73 ± 0.03. • The AI model's average performance significantly outperformed the combined assessment of five radiation oncologists. The AI model achieved an average accuracy of 0.79 and an F1 score of 0.76, whereas the physicians' average accuracy was 0.71 with an F1 score of 0.70.	Training Set:181 Testing Set:48
Ma,2025 (113)	DL(Med3D) Develop a deep learning model that integrates PET/CT images, radiomic features, and clinical data to make predictions using weakly labeled data for the purpose of predicting lymph node metastasis in patients with intermediate- to high-risk prostate cancer.	• On the test set, the multimodal model that integrated clinical parameters, SUVmax, and deep learning features performed best (Model 3, AUC = 0.85), significantly outperforming the model that used only clinical and SUVmax data (Model 1, AUC = 0.74) and the model that relied solely on deep learning features (Model 2, AUC = 0.86). The performance of this multimodal model was significantly superior to that of the MSKCC clinical prediction model (AUC 0.85 vs. 0.79).meterΛ	Training Set:82 Testing Set:34
Wu,2025 (114)	DL(EfficientNetV2) aim to integrate MP-MRI features, peripheral blood lymphocyte functional subpopulation analysis, and clinical variables (such as PI-RADS scores, age, and prostate volume) to more accurately and reliably classify patients into low-, intermediate-, and high-risk groups, thereby aiding clinical decision-making.	• On the independent test set, the multimodal model achieved an AUC of 0.9609, with an F1 score of 0.8671, a sensitivity of 0.9333, and a specificity of 0.8667. • The multimodal model significantly outperformed traditional clinical charts as well as single predictors such as PSA or PI-RADS scores. • Analysis indicates that the MRI score (MS) makes the most significant contribution. If MS is removed from the model, the AUC on the test set drops from 0.96 to 0.70, with its impact exceeding even that of the lymphocyte score (LS) and clinical variables.	Training Set:88 Testing Set:22

## 4 Application of AI in prostate cancer treatment

### 4.1 AI + RP for accurate prediction of postoperative complications

Radical prostatectomy (RP) involves the complete removal of the prostate gland and surrounding tissues that may have been invaded by cancer cells. Depending on specific patient conditions, lymph node dissection and its extent can be selected to achieve curative effects for prostate cancer, so RP is regarded as the primary treatment for localized prostate cancer. In the early stage, open radical prostatectomy (ORP) was performed through a large abdominal incision. Although significant therapeutic effects have been demonstrated with this approach, it is associated with postoperative risks such as hemorrhage, infection, urinary incontinence, erectile dysfunction, and rectal injury. Currently, robot-assisted radical prostatectomy (RARP) combines the minimally invasive nature of laparoscopy with the flexibility and precision of robotic technology. Compared with ORP, RARP is less invasive, with 3D visualization of the surgical field and high precision in instrument movement. It is associated with less blood loss, lower transfusion rates, shorter hospital stays, fewer overall complications, higher nerve preservation rates, accelerated recovery of postoperative erectile function, and reduced biochemical recurrence (BCR) (116, 117)AI uses computer vision to automatically identify and annotate surgical steps. Real-time feedback is provided to surgeons, the workflow is optimized, and potential risks are promptly flagged by AI. Standardized surgical protocols have been established by AI, which can aid novice surgeons in understanding operational norms and reduce subjective differences. A computer vision-based AI algorithm was developed by Khanna (118),which can automatically recognize critical steps in RARP surgery and achieves 92.8% consistency with manual annotation. Zuluaga (119) further employed an AI platform integrated with Video Transformer Network (VTN) technology to accurately label surgical steps and precisely identify critical phases during procedures. Large volumes of automatically annotated surgical videos can be used as training and testing datasets for robotic systems through this platform, allowing the systems to mimic surgeon operations and demonstrate autonomous adaptability. Augmented reality three-dimensional (AR3D) models project 3D virtual models onto robotic consoles by overlaying MRI data. Up to 92% accuracy in cancer localization is achieved by this technology, enabling surgical plans to be adjusted on the basis of precise tumor positioning. Such adjustments are deemed reasonable in nearly 95% of cases, which helps surgeons make improved intraoperative decisions (120).

Currently, the application of AI in RP primarily focuses on predicting postoperative complications using various datasets (**Table 6**). This includes predicting postoperative biochemical recurrence, lymph node metastasis, and recovery from urinary incontinence. By integrating different information models, it is possible to accurately predict and identify the most significant influencing factors. It is evident that the integration of AI with RP has fostered comprehensive technological innovation across preoperative planning, intraoperative navigation, and postoperative prediction. This effectively addresses inherent limitations such as the previous heavy reliance on the lead surgeon’s experience and restricted intraoperative anatomical recognition. However, it also faces specific limitations and shortcomings; For example, models related to RARP may only be compatible with a single brand or model of surgical robotic system; currently, they cannot perform precise analyses that disregard device-specific factors, resulting in high specialization but low transferability; The integration of holographic imaging with intraoperative navigation, which we anticipate, is still in its early exploratory stages. While it can overlay the visibility of superficial anatomical structures, it cannot accommodate complex, individualized anatomical scenarios such as local tumor infiltration, variations in neurovascular bundles, or postoperative scarring. Furthermore, it cannot accurately correct for real-time displacements of anatomical structures caused by dynamic traction, tissue deformation, or instrument compression during surgery, making it prone to navigation errors and unable to meet the precise surgical requirements of high-risk, complex RARP procedures. Training models based on conventional standardized surgical procedures are limited to simple, repetitive operations and cannot address the dynamic decision-making scenarios encountered during high-difficulty RARP, such as unexpected intraoperative hemostasis, adhesion separation, and variations in lymph node dissection—all of which are highly complex and non-routine situations. Furthermore, current AI research on RARP lacks a standardized training dataset. Different studies exhibit inconsistencies in instrument movement stability, procedural fluidity, and adherence to key steps, preventing the establishment of a standardized RARP intelligent quality control and training system. Additionally, there is a lack of long-term prospective validation for postoperative urinary control and sexual function recovery following RARP. there remains a lack of specific clinical evidence to support whether AI-assisted RARP can effectively improve patients’ long-term oncological and functional outcomes, which significantly limits the practical application value of AI technology in the clinical implementation of precise RARP procedures and standardized promotion.

**Table 6:** AI-assisted postoperative risk prediction for RP.

Author, year	AI model and research objectives	Main finding	Data
Kwong,2023 (121)	SEPERA-Based XGBoost Prediction of Unilateral Extracapsular Prostate Invasion (ssEPE) in RARP Patients	• SEPERA achieved an AUROC of 0.80 on the training set and 0.77 on the validation set, both higher than those of logistic regression, the two-class Soeterik model, and the Sayyid plot reference model. • SEPERA achieved an accuracy of 68% (72/106) in predicting difficult cases. • SHAP identified the most important factor influencing ssEPE: the proportion of involved tissue in the prostatic base biopsy.	Training Set:1022 Testing Set:3914
Hao,2022 (122)	A personalized predictive model for postoperative PSM following RARP using univariate and multivariate logistic regression models	• Significant differences were observed between the PSM group and the negative surgical margin (NSM) group in terms of age, PPN, ISUP score, PT, PI-RADS score, tumor location, maximum tumor diameter, T-MRI staging, PSA, and PSAD. • ISUP score, PI-RADS score, and PSA are independent risk factors for PSM following RALP.	Training Set:882
Kawase,2022 (123)	The Kaplan–Meier method and the Cox proportional hazards regression model were used to identify and validate preoperative risk factors predictive of postoperative biochemical recurrence.	• A Gleason score ≥3 is an independent risk factor for biochemical recurrence (BCR) following radical prostatectomy (RARP) in patients with intermediate-risk prostate cancer and an independent preoperative predictor; none of the other indicators were statistically significant and can be used for preoperative risk stratification.	Training Set:1144
Liu,2026 (124)	Univariate and multivariate Cox proportional hazards regression were used to construct a predictive model for postoperative biochemical recurrence following RARP and to identify independent prognostic risk factors.	• The model achieved AUC values ranging from 0.81 to 0.92 on both the training set and the internal validation set, but these values decreased on the external validation set, though they remained at an acceptable level. • The most significant factor identified as being significantly associated with postoperative BCR-free survival was free prostate-specific antigen (fPSA).	Training and Internal Test Set:450 External Test Set:175
Morgan,2025 (125)	Development and validation of a multimodal artificial intelligence model (MMAI) based on digital pathology to predict the risk of biochemical recurrence and distant metastasis (DM) following resection of rectal cancer (RP).	• The MMAI model had a tdAUC of 0.74,outperforming traditional clinical roadmaps and COX models based solely on clinical indicators. • For every 1-standard-deviation increase in the MMAI score, the sub-distribution hazard ratio (sHR) for distant metastasis was 2.17. • After adjusting for all clinical indicators, the MMAI score retained its independent prognostic value (sHR = 2.56).	Training set:1322 Internal Test Set:533
Hakozaki,2023 (126)	Univariate and multivariate logistic regression models identify independent predictors of urinary function recovery at 1 year in patients with postoperative urinary incontinence (UIIAS) following LRP and RARP	• Multivariate logistic regression revealed that two independent protective factors and predictors were both associated with age: patients under 65 years of age were more likely to regain urinary control. • The remaining factors were not significantly associated with urinary control at 1 year post-surgery and were not independent predictors of recovery of urinary control.	Training Set:274
Amparore,2024 (127)	XGBoost; Logistic Regression; Random Forests: Predicting Postoperative Urinary Incontinence After RARP Based on Patient Clinical Information	• XGBoost: AUC = 0.65 (best predictive performance); Logistic Regression: AUC = 0.58; Random Forest: AUC = 0.55. • The top four variables identified by SHAP analysis as having the greatest influence on postoperative urinary incontinence: nerve-sparing surgical procedure, age, digital rectal examination (DRE) results, and total PSA level.	Training Set:227
Li,2023 (128)	Captum, a deep neural network (DNN)-based model, combines multiple preoperative MRI anatomical parameters to predict postoperative urinary incontinence following RARP	• UINet7 achieved an AUC of 0.98, significantly outperforming all traditional models, including KNN, logistic regression, random forests, XGBoost, and SVM. • Captum identified 7 core features, the top 4 of which (age, MUL, PAL, DILAM) were identical to those selected by the traditional LASSO + logistic regression approach.	Training Set:91
Nakamura,2023 (129)	A CNN-based DenseNet169 model integrates intraoperative images with multiple algorithms to predict postoperative urinary control recovery following RARP.	• Image + DL + Integrated ML: AUC = 0.882; external validation set AUC = 0.858 (best result); performance did not improve after incorporating clinical and pathological parameters • The early and delayed urinary continence groups showed statistically significant differences in BMI, history of NADT use, membranous urethral length (MUL), and Gleason score, which are independent predictors of postoperative urinary continence recovery	Training Set:101 Testing Set:30
Nakanishi,2022 (130)	A Study of the Effects of Postoperative Membranous Urethral Length (MUL) and Bladder-Urethral Anastomosis Position (PVUA) on Short-Term Urinary Control Recovery After RARP Using Univariate and Multivariate Logistic Regression Models	• Univariate logistic regression analysis of urinary control recovery at 3 months postoperatively: BMI < 25 kg/m²;preoperative membranous urethral length ≥ 9.5 mm; postoperative membranous urethral length ≥ 9 mm (P < 0.001). • Multivariate logistic regression analysis, after adjusting for confounding factors, identified only two independent predictors of urinary continence recovery at 3 months post-surgery: postoperative MUL ≥ 9 mm and PVUA < 14.5 mm.	Training Set:215
Shao,2023 (131)	ResNet50 was used to evaluate the predictive value of various postoperative cystographic features for the recovery of PPI following RARP.	• AI model performance results: accuracy 92.5%, sensitivity 87.5%, specificity 97.5%. The Grad-CAM validation model identified the core region of the images as being concentrated in the bladder neck. • Comparison of the rapid recovery group versus the slow recovery group: The incidence of anastomotic leakage was significantly lower (14.8% vs. 32.6%);Significantly shorter bladder neck descent (0.74 cm vs. 1.31 cm);Significantly greater bladder neck angle (128.37° vs. 110.78°). • Bladder neck descent: AUC = 0.777; Bladder neck angle: AUC = 0.745.	Training Set:170
Lee,2022 (132)	The Predictive Value of Machine Learning Methods Combined with Patient Clinical Characteristics and Surgeon Objective Performance Measures (APMs) for Predicting Positive Surgical Margins (PSMs) Following RARP	• Full model (clinical factors + APMs): AUC = 0.74, demonstrating the best predictive performance. Model based solely on clinical factors: AUC = 0.72, with performance comparable to the full model. • The strongest predictors, in order, are: extracapsular extension (ECE) > pathological T stage (pT).	Training Set:236

### 4.2 AI + radiotherapy for automatic planning and efficient irradiation

Radiotherapy for prostate cancer can serve either as an adjunct to curative surgery or as a standalone treatment for the disease. It can be categorized into intensity-modulated radiation therapy (IMRT), image-guided radiation therapy (IGRT), stereotactic body radiation therapy (SBRT), and brachytherapy. The integration of AI with radiation therapy for prostate cancer drives precision medicine by combining imaging data to improve the accuracy of tumor target and surrounding tissue delineation; it also optimizes dose distribution to reduce toxicity to critical organs such as the rectum (133).The emergence of AI has further facilitated the realization of precision radiotherapy for prostate cancer. Treatment planning before the initiation of radiotherapy is highly complex and is affected by the proficiency of radiation oncologists. To achieve optimal treatment outcomes, repeated parameter adjustments are required. The core principles of prostate cancer radiotherapy are precise coverage of the tumor target area and maximum protection of surrounding critical organs such as the bladder, rectum, and femoral head. Precise delineation of the target area and normal organs, along with individualized dose modulation, are the two key factors in ensuring treatment efficacy and reducing acute and chronic toxic side effects. Due to the prostate’s unique anatomical location adjacent to multiple sensitive organs, coupled with the high subjectivity, poor reproducibility, and time-consuming nature of traditional manual contouring, conventional dose optimization models struggle to balance the conflicting goals of delivering sufficient radiation to the target area while protecting normal tissues from high doses (134),which significantly compromises the safety and precision of prostate cancer radiotherapy. AI-driven deep learning for precise segmentation, dose prediction, and end-to-end automation has effectively overcome the technical bottlenecks of traditional radiotherapy. It enables efficient, standardized precise contouring of multiple organs, achieving a relative balance between the irradiation doses for the target volume and critical organs. Through literature screening, we found that research focuses on the intelligent contouring of radiotherapy target volumes and critical organs, as well as the automated generation and optimization of radiotherapy plans (**Table 7**). AI has already achieved results that show no significant difference from those of physician-generated plans and segmentations. The results indicate that some models outperform manual methods in the accuracy of delineating at-risk organs (such as the rectum and bladder). Gao,et al. (152) developed a virtual treatment plan (VTP) to evaluate the efficacy and capabilities of treatment planning for SBRT in prostate cancer patients. After multiple refinements, the VTP achieved near-perfect scores in radiotherapy planning, slightly overperforming manually designed plans, which demonstrates the feasibility of its framework. The deep learning-based RTP-Net is capable of accurately contouring tumors and critical organs throughout the body, with accuracy comparable to or even surpassing that of manual contouring. For most tasks, contouring can be completed within two seconds, approaching real-time processing (153). Compared to clinicians, deep learning-based automatic segmentation techniques achieved higher overall satisfaction in contouring clinical target volumes (CTVs) for radiation therapy planning. Although minor adjustments and modifications by physicians are still required, AI-driven automatic segmentation technology saves time, improves delineation quality, and reduces inter-observer variability (135, 154). DeKerf et al. (155) proposed a novel evaluation metric to assess both segmentation and planning model performance in prostate cancer stereotactic SBRT. Deep learning segmentation (DLS) models achieve nearly identical segmentation of the bladder and rectum to that of clinicians, with similarity values close to 1. Although some models perform slightly worse than manual methods in the automatic delineation of prostate cancer treatment fields, the DSC and HD values remain within clinically acceptable ranges. Similarly, AI-generated radiation treatment plans differ very little from manually created ones; 85%–90% can be directly applied in clinical practice, while a small portion requires minor manual adjustments before clinical use. This indicates that artificial intelligence has established a new, reliable method for prostate treatment planning. AI-driven precise delineation of the treatment target and critical organs, along with radiation treatment planning, can ensure that the tumor target receives the full prescribed dose while protecting surrounding normal organs. This significantly reduces variability in treatment practices among different physicians and across different centers, reducing the time required from several hours to just a few minutes, and demonstrates the clinical value and potential of standardized and personalized radiation therapy decisions for patients. However, AI cannot yet operate entirely independently of human intervention due to fundamental limitations. As shown in the table, the vast majority of studies are single-center, retrospective cohort studies with small sample sizes and a lack of external validation sets. Furthermore, most are based on data from specific geographic regions and ethnic groups. This explains why models perform well on internal validation sets but show varying degrees of decline on external validation sets. We need to establish open datasets to standardize image acquisition and contour annotation, covering a large-scale, multi-center, standardized dataset that spans different devices, diverse populations, and complex cases. This will enable the design of prospective randomized controlled trials to clarify the true clinical benefits of AI-assisted radiotherapy. We will develop multimodal fusion segmentation models that deeply integrate CT, MRI, and PET imaging features, focusing on overcoming the challenges of accurately segmenting complex structures such as the prostate, bulb of the penis, and seminal vesicles. At the same time, we will move beyond purely visual modeling tools to develop explainable algorithms grounded in clinical logic, thereby advancing the standardized and end-to-end intelligent application of AI in the field of prostate cancer radiotherapy.

**Table 7:** AI-assisted automation of prostate cancer radiation therapy planning, organ segmentation, and dose control.

Author, year	AI model and research objectives	Main finding	Data
Polymeri,2024 (135)	3D U-Net Optimize existing AI algorithms to enable fully automated contouring of the prostate and surrounding critical organs (the bladder and rectum) in CT images used for radiation therapy planning	• Organ delineation: Prostate alone (184 cases): median DSC = 0.82, median HD = 9.2 mm; Prostate + seminal vesicles (145 cases): median DSC = 0.83, median HD = 11.4 mm.Bladder (335 cases): median DSC = 0.95, median HD = 6.7 mm; • In all tested cases, there were no instances of complete failure to outline the AI; • Outline quality ranking: bladder > rectum > prostate	Total sample:4528 Training set:50%,internal test set:25%.external test set 25%
Arjmandi,2025 (136)	hybrid CNN-ViT model evaluating the dosimetric impact of a deep learning-based automatic contouring model on the delineation of the clinical target volume (CTV) and at-risk organs for prostate cancer radiotherapy. The study also compares the geometric accuracy of this model with that of manual contouring performed by experts.	• With the exception of the rectum, the overlap between the Auto-C+OC and Auto-EC+OC methods was higher than that of the EC-OC method. CTV: AC-OC 91.18 ± 1.65; Bladder: AC-OC 95.46 ± 0.84; Rectum: EC-OC 91.60 ± 2.62; • The irradiation fields and dose levels for target areas and critical organs determined by AI were similar to those planned by senior physicians, with no significant differences between groups. • Contouring time ranged from 25 minutes for manual methods to 30 seconds for AI, representing a speed increase of several dozen times, which significantly reduced the workload for medical staff.	Total sample 104 training set:internal test set:external test set 7:3:1
Dinç,2025 (137)	DirectORGANS Evaluation of the Clinical Feasibility of DirectORGANS for Direct Automated Organ Contour Tracing in CT-Guided Radiotherapy for Prostate Cancer	• Overall, there was no statistically significant difference in organ-at-risk doses between automatic and manual contouring; however, there were statistically significant differences in the CI and HI indices for the prostate target volume. • Conformity Index (CI): Manual contouring 0.98, automatic contouring 0.852; Homogeneity Index (HI): Manual contouring 0.0395, automatic contouring 0.1715; Dose homogeneity was significantly reduced in the automatic contouring group.	Total sample:10
Hemon,2023 (138)	VoxelMorph(Weakly Supervised U-Net Architecture) Feasibility and Efficacy of Deep Learning-Based Deformed Image Registration for Calculating Cumulative Dose in CBCT-Guided Radiotherapy for Prostate Cancer	• DSC mean range: VMorph_Sc (image only): 0.67–0.79; VMorph_Msk (mask only): 0.93–0.98 (optimal); VMorph_Sc_Msk (image + mask): 0.89–0.96 • Prostate: The cumulative doses calculated by all models showed no significant difference from the planned doses, indicating stable dose estimation. However, for the bladder and rectum, the AI models calculated doses that were either higher or lower than the actual doses, resulting in unstable outcomes.	Total sample:23
Kawaguchi,2026 (139)	OncoStudio 2.0(CNN) A preliminary study investigating the dosimetric effects of AI-generated seminal vesicle contours versus manually drawn contours in volumetric modulated arc therapy (VMAT) plans for high-risk prostate cancer.	• There were no statistically significant differences in any DVH parameters among the three groups (manual delineation, automatic delineation after manual adjustment, and automatic delineation without adjustment; the dose difference between AI delineation and manual delineation was less than 1% of the prescribed dose, which is within the clinically acceptable range.	Total sample:15
Kawula,2022 (140)	3D U-Net To investigate the results of automatic organ segmentation in CT images using a 3D U-Net model, compare the differences in radiation therapy plans between manual and AI-generated segmentation, and evaluate the practical impact of dose optimization in radiation therapy plans for prostate cancer.	• DSC/Average HD: Prostate: 0.87 ± 0.03/1.6 ± 0.4; Bladder: 0.97 ± 0.01/0.95 ± 0.2; Rectum: 0.89 ± 0.04/1.4 ± 0.7 • The dose coverage of the AI-planned target areas generally met the criteria, although it was slightly lower than that of the manual plan; however, this did not result in excessive radiation doses to the rectum or bladder (vital organs), and there was no statistically significant difference compared to the manual plan.	Total sample:69
Langner,2025 (141)	iAIC(nnU-Net), A Comparison of Open-Source and Commercial Segmentation Models in Terms of Contouring Accuracy, Processing Speed, and Clinical Usability for Online Adaptive MR-Guided Radiotherapy of the Pelvic Region Using the Same Training Dataset	• Target area + organs at risk: iAIC mean DSC = 0.87, mean HD95 = 11.8 mm; cAIC mean DSC = 0.83, mean HD95 = 10.2 mm. • 2. DSC for each anatomical structure (iAIC:cAIC): Bladder: 0.97:0.97; Prostate: 0.89:0.86; Rectum: 0.91:0.91; Seminal vesicles: 0.82:0.76. HD95 (iAIC:cAIC): Bladder: 5.6:6.0; Rectum: 17.3:20.6;	training set:47 internal test set:20
Nachbar,2024 (142)	UNet, Train and validate a fast, accurate deep learning-based automatic segmentation model to enable the automatic delineation of anatomical structures relevant to prostate cancer radiotherapy on MRI scans	• Median maximum DSC value: bladder 0.97. Median minimum value: penile bulb 0.73; median maximum 95% HD value: rectum 6.9 mm, median minimum value: bladder 2.7 mm; • The median time for AI to automatically complete the delineation of all structures was 152 seconds, ranging from 121 to 198 seconds, which is significantly faster than manual delineation.	Total sample:47
Schmidt,2026 (143)	Ethos A comparison of the accuracy and efficiency of pelvic organ segmentation for prostate cancer using three methods—manual segmentation (MD), fully automated AI segmentation (AI), and hybrid AI-assisted segmentation with manual correction (MD+AI)—on pCT and hCBCT scans.	• High-precision organs (bladder, bilateral femoral heads): Median DSC: 0.95–0.96; Median HD95: 1.88–2.17 mm. MD+AI further reduces inter-rater variability. • Medium-precision organs (prostate, rectum): Median DSC: 0.92; Median HD95: 2.22–2.62 mm. • Low-precision organs (seminal vesicles, corpus spongiosum): Median DSC: 0.76–0.83; Median HD95: 3.01–3.41 mm; these structures exhibit the greatest delineation variability among all organs.	training set:46,internal test set:38
Shen,2023 (144)	CUNet (3D U-Net) To evaluate the accuracy and clinical applicability of CUNet for the automatic contouring of the CTV and OARs in prostate cancer radiotherapy.	• The mean DSC for the CTV was 0.84 ± 0.05, and the mean 95HD was 5.04 ± 2.15 mm. • organ at risk (OAR): Bladder (highest): DSC 0.913 ± 0.078, 95HD 2.462 ± 3.984 mm; Rectum (lowest): DSC 0.783 ± 0.032, 95HD 6.278 ± 2.275 mm • There were no significant differences between the radiotherapy plans generated by automatic contouring and the clinical standard plans; dose distribution consistency was good, and automatic contouring of a single patient’s CT images took <15 seconds, whereas manual contouring typically requires 10–20 minutes.	training set,+validation set:195 internal test set:28
Wen,2024 (145)	DeeplabV3+, Unet++, 3D Unet Develop a fully automated contouring model based on deep learning, tailored for two major clinical scenarios: radical radiotherapy for prostate cancer and postoperative radiotherapy, to enable automatic target segmentation of pelvic lymph nodes (CTVn) and prostate tumors/postoperative tumor beds (CTVp).	1. Postoperative radiotherapy model: VD for CTVn = 0.86, VD for CTVp = 0.79; HD95 for the combined target volume = 5.53 mm. Radical radiotherapy model: VD for CTVn = 0.85, VD for CTVp = 0.84; HD95 for the combined target volume = 4.00 mm. 2. Comparison of Contouring Accuracy Between AI and Less Experienced Physicians Postoperative radiotherapy model: CTVn (pelvic lymph nodes): VD 0.86:0.83; CTVp (prostate tumor bed): VD = 0.79:0.73; Radical radiotherapy model: CTVn (pelvic lymph nodes): VD 0.85:0.82; CTVp (prostate tumor bed): Both AI and physician VD were 0.84.	training set,+validation set:167,internal test set:30
Arjmandi,2026 (146)	Limbus Contouring Software(U-Net) To evaluate the effectiveness of a commercial AI-assisted contouring tool in reducing intra- and inter-observer contouring variability in prostate radiotherapy. To analyze the dosimetric implications of geometric contouring differences and determine the clinical value of different contouring methods.	• Compared to purely manual contouring, AI-adjusted contours showed a significant increase in DSC and a marked decrease in HD across all anatomical structures. The CTV DSC increased from 0.86 ± 0.03 to 0.90 ± 0.08, while the HD95 decreased from 6.3 ± 1.8 mm to 2.6 ± 1.1 mm; • AI-assisted contouring significantly reduced the variation in contouring between the two radiologists. The CTV DSC increased from 0.78 ± 0.11 to 0.89 ± 0.09; the HD95 decreased from 15.9 ± 2.1 mm to 4.7 ± 2.2 mm; and for the rectum, the HD95 decreased from 20.1 mm to 3.6 mm.	Total sample:15
Chung,2025 (147)	RapidPlan(v15.6.06) For 10 different tumor sites, we developed and validated multiple clinically applicable, fully automated KBP models to enable the fully automated generation of VMAT treatment plans without human intervention, and validated their dosimetric performance.	• For the prostate cancer and prostate cancer with lymph node metastasis models, the radiation dose to surrounding normal tissues was kept within safe limits in 95% of cases for both models, and the average dose in the primary treatment area reached 96.9% and 98.6% of the prescribed standard, respectively. • 83% of the treatment plans generated for the prostate cancer-only model were ready for use without modification; 98% of those for the prostate cancer with lymph node metastasis model were ready for use.	training set:132 validation set:58
Bolten,2025 (148)	RLS Male Pelvic v2.0.0+RSL-Prostate-6000 To evaluate the clinical feasibility of a machine learning-based, one-click, fully automated radiation therapy planning workflow in the treatment of prostate cancer	• CTV: The volume of the CTV automatically contoured by AI was significantly smaller than that contoured manually: 47.1 cm³ vs. 62.6 cm³. Inter-rater agreement for manual contouring was DSC = 0.90 ± 0.04 and HD = 0.98 ± 0.33; for AI contouring, DSC = 0.86 ± 0.04 and HD = 1.27 ± 0.45. • Overall, the deviations between AI-contoured and manually contoured volumes remain within clinically acceptable inter-observer variability ranges, particularly for the bladder and rectum, where they are significantly smaller than those of manual planning, and the stability of AI re-contouring results is good.	Total sample:5
Gaudreault,2025 (149)	CNN+Adam+AMSGrad By incorporating deep learning methods, this approach predicts the monitoring units (MUs) for each control point in VMAT radiotherapy for prostate cancer, thereby generating an AI-based radiation treatment plan (AI-RTPlan).	• Compressing three-dimensional radiation therapy dose maps into two-dimensional (2D) images yields the best results: for 93% of patients, AI-generated treatment plans are as effective as manually generated plans in terms of treatment outcomes and organ protection, with minimal deviations in radiation dose.	training set:220,validation set:40,internal test set:42
Kadoya,2023 (150)	HD-U-net To evaluate the dosimetric accuracy and clinical utility of the deep learning-based AIVOT prototype system in generating executable VMAT radiation therapy plans for prostate cancer patients; to verify whether the system can achieve fully automated radiation therapy planning without human intervention	• Implementable plan (deliDose) vs. clinical plan (cliDose) (key results):Mean absolute dose difference for all DVH parameters in the target region: 1.32 ± 1.35; mean absolute dose difference for at-risk organs: 2.08 ± 2.79%. • For tumor lesions, the radiation coverage and dose intensity of the AI plan are essentially consistent with those of the manual plan, ensuring that the tumor is adequately irradiated;	training set:55, internal test set:13
Khalifa,2024 (151)	An Atlas-Based Model for Automated Machine Learning Planning To evaluate the efficacy of machine learning (ML)-based automated adaptive radiation therapy planning in online MRI-guided adaptive radiation therapy for prostate cancer.	• Target Volume (CTV/PTV) Performance: The doses to the CTV and PTV met the clinical prescription requirements, with a compliance rate of 100% for both groups; no underdosing issues were observed in the ML planning target volume, and the tumor irradiation dose was adequate and met the requirements. • Overall planning quality parameters: Conformal Index (CI): ML 0.828 ± 0.023, Clinical 0.824 ± 0.036; Homogeneity Index (HI): ML 1.026 ± 0.002, Clinical 1.040 ± 0.003;	training set:46,internal test set:38

### 4.3 AI facilitates the mining of therapeutic targets and biomarkers

Significant challenges are posed to the identification of therapeutic targets, development of pharmaceuticals, and evaluation of drug safety and efficacy by the heterogeneity of prostate cancer and the structural complexity of its genome and proteome. Protracted timelines, high costs, and low efficiency result from these challenges. Artificial intelligence (AI) can identify features beyond human perception and execute extensive repetitive tasks, thereby accelerating the screening of therapeutic targets and the investigation of mechanisms in prostate cancer treatment. P-NETs constitute a biology-based deep learning model; by integrating extensive genomic data, molecular alterations associated with prostate cancer progression and treatment resistance can be elucidated. MDM4 has been revealed as a potential therapeutic target through this model, which provides novel insights for biological research on prostate cancer (156). Multiple machine learning approaches were integrated by Wang,et al. (157) to screen optimal diagnostic and prognostic prediction models. The algorithmic integration of multiomics data was employed to evaluate associations between gene expression and immune infiltration. In combination with machine learning, POLD1 was identified as a novel biomarker linked to prostate cancer metastasis, which offers promising therapeutic avenues for metastatic disease. The MIEC-SVM model was utilized by researchers to discover the novel AR antagonist C2. This antagonist has significant inhibitory effects on AR signaling, suggesting that it has potential for development as a novel prostate cancer therapy (158). Bruzzese (159) used three machine learning models based on genomic and clinical indicators to screen for biomarkers predictive of post-treatment recurrence; NETO2 was retained in all feature combinations, indicating that this gene is a key molecular biomarker for predicting prostate cancer recurrence.

In the fields of biomarker screening and drug development, the continuous improvement of artificial intelligence’s ability to analyze and integrate large amounts of data has undoubtedly made the screening of targeted biomarkers for prostate cancer increasingly innovative and intelligent, driving research into personalized clinical treatment. However, a unique limitation of biomarker screening is that models can only identify specific genes associated with disease progression, but they lack insight into the specific mechanisms of action and lack validation through wet lab experiments, which consequently leads to a high rate of false positives in target screening. Lin (160) trained various machine learning models using public databases and found that PCNA expression in prostate cancer tissue is significantly higher than in normal tissue; in high-risk patients with a Gleason score >7, PCNA protein expression is significantly upregulated; and the accuracy of PCNA as a therapeutic target was validated through *in vitro* and *in vivo* experiments. Only through the integration of AI-based computation and wet lab validation can we understand the specific mechanisms and the true effects of targets, thereby providing patients with safer and more effective treatment options and improving their quality of life and prognosis.

## 5 Application of LLMs in feature extraction and prostate cancer knowledge popularization

Beyond AI models for prostate cancer diagnosis and treatment, text-generating large language models (LLMs), such as ChatGPT and DeepSeek—representing major breakthroughs in AI technology—have entered the public eye and rapidly gained popularity. LLMs can efficiently parse unstructured electronic health records to enable automatic summarization, key information extraction, format standardization, and cross-text conversion, accurately capturing core metrics such as PSA levels, Gleason scores, PI-RADS scores, tumor staging, and capsular invasion. Di Palma et al. (161) compared the ability of multiple LLMs to convert free-text from unstructured prostate MRI reports into a standardized, searchable structured data format. The average accuracy of the five models generally exceeded 96%, with DeepSeek-R1-Llama3.3 (98.6 ± 2.1%) performing the best, making almost no errors; All models achieved 100% extraction of PSA density, prostate volume, and three-dimensional dimensions; however, differences in physicians’ writing styles resulted in an average score variance of approximately 5% across models, with DeepSeek-R1-Llama3.3 demonstrating the highest stability when processing reports from different physicians. Jo et al. (162)developed a fully automated AJCC staging system for postoperative pathology reports based on the information extraction capabilities of LMMs. On the internal test set, the system achieved an accuracy of 0.973 and an F1 score of 0.986, with F1 scores approaching 1 for M staging, SVI, lymph node metastasis, and N staging; On the external validation set, the accuracy was 0.938 and the F1 score was 0.968, with F1 scores of 1.000 for lymph node metastasis, N staging, number of submitted lymph nodes, perineural invasion, SVI, and T staging. It is worth noting that the proportion of secondary Gleason scores was the weakest indicator on both the internal and external validation sets, which may be attributed to errors in converting text percentages. The performance of the fully automated AJCC classification achieved an F1 score of 0.930 on the internal test set and 0.833 on the external test set. Cutting-edge research is focused on developing models capable of processing integrated data that combines text, imaging, pathology, and genomic data. Using language models as the central hub, these models link imaging features with textual conclusions to build integrated diagnostic models. For example, Prost-LM is a multimodal large language model specifically designed for prostate cancer. It embeds MRI image features, quantified PSA metrics, and free-text clinical reports (such as physician descriptions and radiological opinions) into a unified semantic space, enabling cross-modal deep reasoning to enhance diagnostic accuracy. This approach effectively addresses clinical challenges posed by high tumor heterogeneity and the difficulty of integrating multi-source information (163). Shiet,et al. (164)evaluated the practical efficacy of GPT-4 in automatically generating prostate biopsy recommendations based on MP-MRI and clinical data; GPT-4 helped 20.8% (190/912) of patients avoid unnecessary biopsies; Expert evaluations of GPT-4 report quality—covering completeness, comprehensibility, practicality, personalization, and compliance—showed average scores of ≥4.6, with over 90% of reports rated as excellent. LLMs can translate specialized medical terminology into plain language to create easy-to-understand educational content explaining key points regarding examinations, treatments, rehabilitation, and follow-up visits. This has been a hot research area for LLMs in prostate cancer in recent years (**Table 8**). In addition, LLMs support text simplification and summary rewriting capabilities, offering the potential to generate high-quality, accessible summaries at scale. Rinderknecht (173) conducted a large-scale evaluation of ChatGPT-4’s overall performance in generating lay-language summaries for prostate cancer research papers, Overall, the lay summaries generated by ChatGPT-4 outperformed human-written summaries across the board in terms of readability, factual accuracy, and word count compliance. Among the three categories of literature—basic research, clinical research, and translational research—the quality of AI-generated summaries was higher than that of human-written summaries. Notably, basic research texts tend to use simpler vocabulary. Based on the results, the mainstream LLMs currently available on the market perform excellently in terms of accuracy, comprehensibility, and patient trust. They are capable of addressing routine questions regarding prostate cancer diagnosis and treatment as well as providing public health education; however, for critical issues, physician assistance is still required. Nevertheless, some studies evaluate the performance of only a single model and lack comparisons with texts authored by senior experts; Information sources are unclear; without explicit emphasis, sources are not cited. Even when sources are cited, some models have not been updated with the latest diagnostic and treatment guidelines, which could mislead patients. Most importantly, the issues of model “hallucinations” and output reliability require clinical validation to ensure the accuracy of feature extraction (174). Weakness in processing long texts: Given the long follow-up periods and voluminous medical records associated with prostate cancer patients, general-purpose LLMs are prone to omitting key information and producing logical inconsistencies, resulting in a significant drop in accuracy when analyzing complex medical histories involving multiple recurrences and lines of treatment over many years (175).Future research on large language models (LLMs) should focus on advancing the practical implementation of large-scale Retrieval-Augmented Generation (RAG) technology, linking models to authoritative guidelines and high-quality literature databases to ensure that model outputs are verifiable. Currently, responses from general-purpose large language models tend to be broad and cover various types of cancer, lacking specialized models. By building a disease-specific model for prostate cancer based on these large language models, we can improve both relevance and accuracy.

**Table 8:** Applications of LLMs in prostate cancer public outreach and education.

Author, year	LLMs	Research objective	Main findings
Geanta,2024 (165)	ChatGPT 3.5,CoPilot,Gemini	Compare the performance of various large language models against Romania’s official Guide for Prostate Cancer Patients in disseminating public health information about prostate cancer from multiple perspectives.	• Overall composite score: ChatGPT 3.5 > CoPilot > Gemini • Individual evaluation results: Accuracy: ChatGPT > CoPilot > Gemini = Guide; Timeliness: ChatGPT > CoPilot > Guide > Gemini; Comprehensiveness: ChatGPT > CoPilot > Gemini = Guide; Clarity: ChatGPT and CoPilot performed similarly, both outperforming Gemini and the Guide
Gibson,2024 (166)	ChatGPT-4	Comprehensively evaluate the quality, accuracy, safety, appropriateness, practicality, and readability of the responses generated by ChatGPT-4 to common questions asked by prostate cancer patients. Determine whether ChatGPT-4 can serve as a reliable and safe patient education tool in the field of prostate cancer.	• Average score for readability: 79.44%; average score for content quality: 13.88, rated as “Good”; • Evaluations by urology specialists indicate that average scores across all dimensions are above the median; the content is well-aligned with patient needs and poses an extremely low risk of information security breaches. • ChatGPT-4’s responses are generally difficult to read, with an average reading level equivalent to that of 15- to 17-year-olds, far exceeding the 6th to 8th grade level recommended for patient-oriented health education.
Hao,2025 (167)	RadOnc-GPT(GPT-4)	To evaluate the actual effectiveness of a model-assisted system for generating online consultation responses for prostate cancer patients, compared to clinical healthcare providers.	• Emotional Tone: The model’s content completeness and accuracy are roughly on par with those of healthcare professionals, though AI performs better on simple tasks such as interpreting test results and arranging medical procedures. Linguistic Logic: RadOnc-GPT: 92.41% of responses used general, neutral language, with content tending to be vague; healthcare professionals’ responses were 70.25% neutral, while 29.11% were highly relevant to patients’ inquiries and more targeted. • Human baseline time spent: With RadOnc-GPT assistance, nurses saved an average of 5.2 minutes per message, and doctors saved an average of 2.4 minutes.
Litt,2026 (168)	Claude Opus 4.1	Developing OncoEducate, a clinician-supervised generative AI tool designed to create standardized, easy-to-understand educational materials on diagnosis and treatment for urological cancer patients.	• Healthcare professionals rated the model’s practicality, patient-friendliness, and content accuracy at 6 or higher; patients gave it a score of 7, and the vast majority accepted AI-assisted health education. • 65% of patients were able to correctly understand their treatment; compared to previous studies, where recognition rates ranged from only 19% to 31%, this represents a significant increase in understanding
Luo,2026 (169)	DeepSeek R1,ChatGPT-4o	Compare the ability and quality of responses from DeepSeek R1 and ChatGPT-4o when answering questions related to radiation therapy for prostate cancer in both Chinese and English.	• Overall Evaluation: In the Chinese environment, DeepSeek achieved a perfect score (5 points) on 33 questions, accounting for 75.76% of the total, while ChatGPT achieved a perfect score on only 36.36% of the questions; in the English environment, ChatGPT achieved a perfect score on 66.70% of the questions, slightly higher than DeepSeek’s 54.55%. • Results by Question Type: In the Chinese environment, for Basic Knowledge: Patient Education: Treatment and Follow-up, DeepSeek achieved 76.92%: 66.67%: 81.82%; ChatGPT achieved 38.46%; 33.33%: 36.36%.In the English environment: DeepSeek 69.23%: 88.89%: 63.64%; ChatGPT 30.77%: 55.56%: 54.55%
Stenzl,2025 (170)	ChatGPT 4.0	To evaluate the overall quality of ChatGPT 4.0’s responses to prostate cancer-related questions and compare them with content curated by professional medical editors.	• Overall Evaluation: For the vast majority of questions, doctors believed that ChatGPT’s answers were at least as good as—and in some cases even better than—the content compiled by medical editors. Across all questions, doctors unanimously agreed that the AI’s answers were clearer. Readability: The content from both sources was at a college-level reading difficulty, with similar readability. • Results by Question Difficulty: - Introductory science popularization: There was little difference between AI responses and content from professional medical websites. - Intermediate difficulty: Content from medical websites was slightly superior in terms of accuracy and completeness. - Highly specialized questions and cutting-edge controversial topics: AI responses were generally better overall, and medical professionals also found the content to be comprehensive and reliable.
Trapp,2025 (171)	ChatGPT-4,ChatGPT-4o,Google Gemini, Microsoft Copilot, Anthropic Claude AI	By integrating the perspectives of clinicians and patients, this study evaluates the practical capabilities and existing limitations of mainstream large language models (LLMs) in patient education for those undergoing radiation therapy for localized prostate cancer, and verifies the feasibility of using LLMs as tools for patient education.	• Text readability assessment: Gemini > Claude AI > ChatGPT-4 > Copilot > ChatGPT-4o; readability improved significantly when ChatGPT-4o was instructed to use plain language. • Clinician Evaluation: All models scored highly (4.2–4.7 points), and their responses were on-topic; ChatGPT-4o was the only model with no errors in evaluation. • Patient Evaluation: 94% of patients found the responses easy to understand; 86% felt there was minimal use of technical jargon and that the content was accessible; 89% believed the information was accurate and relevant to the topic of prostate cancer radiation therapy;
Wang,2025 (172)	GPT-4o,Gemini 1.5 Pro,Kimi AI,Doubao	A comparison of the quality differences in the generation of bilingual (Chinese-English) prostate cancer teaching lesson plans produced by four mainstream large language models versus a senior urology professor.	• Overall Evaluation: GPT-4o scored the highest for both Chinese and English lesson plans; senior experts scored significantly higher than all AI models for Chinese lesson plans; GPT-4o scored the highest for English lesson plans. • Quality of Chinese lesson plans: Human experts > GPT-4o ≈ Gemini 1.5 Pro > Kimi AI ≈ Doubao • Quality of English lesson plans: GPT-4o ≈ Human experts ≈ Gemini 1.5 Pro > Kimi AI ≈ Doubao

## 6 Challenges and prospects for AI models in prostate cancer

Artificial intelligence (AI) is driving the healthcare sector toward precision, personalization, and efficiency at an unprecedented pace. Its application in the diagnosis and treatment of prostate cancer is regarded as a major innovation. Many AI models have been applied throughout the entire process of prostate cancer diagnosis and treatment, focusing primarily on early screening, improving detection efficiency and diagnostic accuracy, optimizing treatment strategies, and predicting prognosis following different treatments—with accuracy levels that rival or even surpass those of specialist physicians. This has reduced the workload on physicians, minimized variability among practitioners, and consequently lowered the risk of patients being misdiagnosed or overtreated. However, there is a significant gap between the high volume of AI models published for prostate cancer diagnosis and treatment and the number of models actually integrated into clinical workflows. This highlights the core contradiction between rapid improvements in technical performance and barriers to clinical translation. To advance AI from the pages of academic journals into clinical practice, it is essential to understand and overcome these limitations.

At the data level, the ultimate performance of deep learning models is highly dependent on large-scale training datasets validated against gold standards; However, whether it is lesion segmentation and PI-RADS scoring on MRI or Gleason pattern annotation on WSI, these tasks require senior specialists to spend a significant amount of time to complete. Even within the same institution, inter-rater agreement for PI-RADS scoring among different radiologists is only moderate to good, and inter-pathologist agreement for Gleason scoring on the same slide is also low. Prostate needle biopsy samples only about 1% of the prostate volume, and systematic sampling can result in false negatives. This means that the model’s performance ceiling is constrained by data quality from the outset. Data heterogeneity severely limits the model’s ability to generalize across centers. Differences in MRI equipment, field strength, and sequence parameters across medical institutions, as well as unstable factors in pathology—such as WSI tissue fixation time, staining reagents, and section thickness—can cause models that perform excellently in a single-center setting to experience a significant decline in performance in real-world, multi-center environments (176). In addition, there is a lack of representation across different populations and racial groups. Currently, the largest publicly available datasets primarily come from white populations in Europe and the United States, with very few including Asian or African American men. The lack of representation in these models may lead to systemic bias, resulting in higher rates of missed diagnoses. Finally, data privacy regulations strictly limit cross-institutional data sharing. Existing research has adopted federated learning (FL) models, enabling institutions to train local models using their own private training datasets without sharing raw data, which remains securely stored on-site. The parameters of the trained models are aggregated via secure transmission protocols. Under strict privacy protection, this approach achieves “knowledge sharing” and “collaborative model development,” thereby partially addressing data silos and compliance barriers in the healthcare sector (177, 178). The primary task for future research at the data level is to establish a unified standard for the collection and annotation of prostate cancer imaging and pathology data. This involves developing specific acquisition protocols for MP-MRI parameters, as well as standards for slide staining and scanning tailored for AI training. Based on these unified standards, a large-scale, standardized reference database spanning different ethnicities, regions, and manufacturers should be constructed. Concurrently, we must further develop federated learning protocols to enable multi-center collaborative training while strictly protecting patient privacy (179). If a model demonstrates consistent performance across test sets from different centers adhering to the same standards, it indicates the model is suitable for clinical use, thereby accelerating its clinical translation.

From a technical perspective, the primary issue is the “black box” nature of AI models themselves. The lack of transparent explanations for the internal decision-making logic of these models severely limits clinicians’ trust and adoption. This is also the main reason why AI models have failed to gain widespread implementation. Explainable AI can be divided into *post-hoc* explainability—commonly used to adapt CNNs and Transformers—and native explainable models that incorporate logic directly into the model itself. Advancements in interpretable AI will be a key breakthrough in gaining clinical trust and represent a major current research focus in the field. Taking SHAP—currently the most popular method—as an example, it calculates the contribution of each input feature to the final result, thereby identifying which specific indicators drive the AI’s classification of high-risk cases. A multicenter study used T2WI and ADC maps to accurately distinguish between low-to-moderate-risk (GG ≤ 2) and moderate-to-high-risk (GG ≥ 3) prostate cancer, achieving AUCs of 0.75 and 0.73 in the test and validation sets, respectively. Of the ten radiomics features identified via Affinity Propagation, seven were derived from the ADC sequence and three from T2WI. SHAP analysis identified the most important feature as ADC-GLRLM-Run Length Non-Uniformity (gray-level run length non-uniformity in the ADC image); higher values indicate greater heterogeneity within the tumor tissue, suggesting high invasiveness; conversely, the second most important feature was ADC-GLRLM-Run Entropy (gray-level run entropy in the ADC image), where lower values are associated with lower invasiveness (180). Altıntaş (181) et al. were the first to combine systemic inflammatory markers with clinical parameters and radiomics to predict the “gray zone” of PI-RADS=3 lesions using six machine learning models; Random Forest (RF) was the optimal model with an AUC of 0.92 and an accuracy of 0.86. SHAP analysis revealed that the top two features contributing most to the prediction of malignancy in PI-RADS=3 lesions were still related to ADC images: tumor ADC value and tumor ADC/contralateral ADC ratio, respectively; the third was PSA density (PSAD); Among inflammatory markers, the Systemic Inflammation Index (SII) and the Neutrophil-to-Lymphocyte Ratio (NLR) demonstrated significantly higher accuracy in predicting malignancy compared to clinical indicators (total PSA, age, and lesion size), providing new predictive biomarkers for clinical practice. Multiple SHAP studies have confirmed the importance of key features in ADC images for high-risk diagnosis in prostate cancer, a finding consistent with actual clinical practice. This enhances clinicians’ trust in AI tools and represents a crucial step in moving AI from a “black box” toward clinical implementation. In patients with bone-metastatic prostate cancer, a random forest model constructed using four factors—PSA nadir, time to PSA nadir (timePSA), number of bone metastases, and lactate dehydrogenase (LDH)—was interpreted using SHAP, confirming that these variables are independent risk factors for rapid progression to metastatic castration-resistant prostate cancer (mCRPC). The model achieved AUC values of 0.946 and 0.927 in the training and validation sets, respectively (182). By quantifying the contribution of each feature to the model’s predictions, SHAP can identify the biological features most critical for disease classification or prognosis from sequencing data, thereby aiding in the discovery of novel diagnostic biomarkers. Ramírez-Mena (183) and colleagues constructed a highly accurate and interpretable model for predicting prostate cancer based on the TCGA-PRAD database. Through differential expression analysis, GO functional enrichment, literature integration, and clustering, they ultimately identified 47 genes associated with prostate cancer (47-PCa-Genes). Among the models tested, the random forest (RF) combined with a downsampling model performed best, with a sensitivity of 0.90, specificity of 0.92, and AUC of 0.91. The model was validated on four external datasets, and SHAP analysis revealed that upregulation of DLX1 and HPN, and downregulation of FGFR2, MYL9, and CNN1, led to a classification result favoring prostate cancer, whereas the opposite pattern indicated normal tissue. Using explainable artificial intelligence (XAI) combined with a dual-gate field-effect transistor (FET) biosensor to detect potential biomarkers in urinary exosomes, SHAP analysis indicated that TMEM256 is the key determinant for PI-RADS 3 patients. Combining AI with the selected biomarkers yielded an AUC of 0.93 and a sensitivity of 0.941. This marks the first time that a combination of biosensors, multiple urinary exosome biomarkers, and XAI has been used for prostate cancer screening. Without the need for a biopsy and using only a urine sample, this approach improved the diagnostic accuracy for PI-RADS 3 from 30.4% to 66.7% (8). Prostate cancer often progresses to castration-resistant prostate cancer (CRPC) 2–3 years after androgen deprivation therapy (ADT); however, there are currently no biomarkers available for detecting CRPC. Research suggests that a class of drug-tolerant persistent cells (DTPs) exists in prostate cancer, which is a key factor in the development of drug resistance. Through proteomics screening, proteins stably and highly expressed in DTP cells were identified, including GPX4, NDUFS4, PRDX5, and TXNRD2, and their validity as predictive biomarkers for CRPC was validated. Machine learning was then used to construct classification and time-prediction models; the RF model achieved an AUC of 0.958 for distinguishing benign from malignant tumors in the tissue cohort; with an AUC of 0.904 on the external test set; the XGBoost model achieved an AUC of 0.988 for distinguishing BPH, LPCa, and CRPC in the serum cohort, and an AUC of 0.917 on the independent validation set; the time prediction model showed that the levels of these four proteins were significantly negatively correlated with TPC (time from diagnosis to CRPC). SHAP analysis indicated that GPX4 is the key gene for BPH and CRPC, while the largest contribution to LPCa comes from NDUFS4 (184).However, the generated heatmaps and contribution values only answer “where is important,” not “why it is important.” AI for medical image explainability suffers from insufficient algorithmic robustness—leading to the misidentification of image edges as actual lesions—as well as a lack of dedicated quantification standards, label noise, the absence of pixel-level fine-grained annotations, and a lack of regulatory guidelines for model deployment. This also indicates that our future research direction should shift from *post-hoc* explainability to interactive visualization platforms, allowing physicians to view the AI’s reasoning basis for suspicious areas. Such human-machine interactive explainability holds far greater clinical value than static heatmaps. We should accelerate the establishment of a supporting ethical and regulatory framework to facilitate the implementation of explainable AI (185).

However, the vast majority of studies focus on single tasks: either lesion detection and PI-RADS classification on MRI, Gleason grading of WSI pathology images, or prognostic prediction based on clinical variables. These models operate in isolation and cannot form a unified decision support system. The multimodal models for diagnostic assistance discussed earlier merely fuse imaging, pathology, and clinical data. However, incomplete multimodal data for some patients can lead to biased test results. Furthermore, existing models are almost exclusively cross-sectional analyses, overlooking the trajectory of PSA over time and the dynamic changes in lesions—time-series information that is crucial for predicting disease progression. Furthermore, in real-world clinical settings, physicians must integrate multidimensional data—including imaging, pathology, PSA dynamics, genomic information, family history, and comorbidities—to make informed judgments. However, multi-omics technologies are often overlooked in multimodal fusion. In reality, AI can learn the relationships between vast amounts of genomic sequences and gene expression data to predict the regulatory patterns of a given sequence across different cell types. In the context of precision diagnosis and treatment for prostate cancer, combining metabolomics, transcriptomics, and radiopathology, and performing deep integration of multi-source data through machine learning and deep analysis algorithms, can comprehensively reveal the complex nature and heterogeneous characteristics of prostate cancer. This approach addresses the limitations in sensitivity and specificity of single diagnostic methods, thereby establishing a comprehensive research and application framework for multimodal intelligent auxiliary diagnosis and personalized precision treatment of prostate cancer.

At the clinical translation stage, the most pressing issue is a severe lack of external validation and prospective studies. The vast majority of studies employ single-center, retrospective designs, often using curated datasets that exclude complex cases that significantly impact model accuracy. Such “idealized” datasets fail to reflect the distribution of real-world cases, and model performance often declines significantly when deployed in practice. Unclear regulatory approval processes and the definition of legal liability are also key barriers to translation. There are no clear legal regulations worldwide regarding liability for misdiagnoses. Meanwhile, some physicians may develop automation bias, over-relying on AI and abandoning their own clinical judgment; others may resist using AI out of concern for professional autonomy. Moving forward, we must reduce the number of retrospective studies and primarily conduct multicenter, randomized controlled trials to quantify the true clinical benefits of these models. Detailed guidelines for liability in AI-related medical malpractice must be established, while clearly defining AI’s role as high-throughput initial screening, alerting for suspicious cases, and quantitative feature extraction—not as a replacement for urologists, radiologists, or pathologists. A standardized human-machine collaborative workflow—”AI-driven bulk initial screening and triage followed by specialist review and confirmation”—should be established to free physicians from repetitive tasks while maintaining the safety baseline of clinical diagnosis (186).

AI technology has demonstrated standardized and highly reproducible advantages in specific scenarios such as MP-MRI interpretation for prostate cancer, Gleason grading, radiation therapy contouring, and prognosis prediction, effectively alleviating clinical pain points associated with significant inter-observer variability and overtreatment in traditional diagnosis and treatment. However, at this stage, the vast majority of models remain in the laboratory research phase, constrained by multiple factors including data bias, the “black box” nature of AI (lack of interpretability), insufficient generalization capabilities, a lack of clinical validation, and incomplete regulatory and ethical frameworks. In the future, AI for prostate cancer will inevitably transition from single-task, single-modality, single-center retrospective algorithms to a comprehensive system featuring multi-modal foundational large models, federated multi-center collaboration, native interpretability, prospective clinical validation, and integration into the entire diagnostic and treatment workflow; Technologically, the focus will be on human-machine collaborative positioning, while regulatory efforts will improve the full lifecycle management of software medical devices. Ultimately, this will achieve a fully intelligent, closed-loop process spanning screening, diagnosis, risk stratification, personalized treatment, intraoperative navigation, and long-term follow-up. This will truly advance the precision diagnosis and treatment of prostate cancer, reducing the burden on the healthcare system while improving long-term survival outcomes for patients across diverse populations.
